# A *MUTYH* germline mutation is associated with small intestinal neuroendocrine tumors

**DOI:** 10.1530/ERC-17-0196

**Published:** 2017-06-20

**Authors:** Jan P Dumanski, Chiara Rasi, Peyman Björklund, Hanna Davies, Abir S Ali, Malin Grönberg, Staffan Welin, Halfdan Sorbye, Henning Grønbæk, Janet L Cunningham, Lars A Forsberg, Lars Lind, Erik Ingelsson, Peter Stålberg, Per Hellman, Eva Tiensuu Janson

**Affiliations:** 1 Department of ImmunologyGenetics and Pathology and SciLifeLab, Uppsala University, Uppsala, Sweden; 2 Department of Surgical SciencesExperimental Surgery, Uppsala University, Uppsala, Sweden; 3 Department of Medical SciencesEndocrine Oncology, Uppsala University, Uppsala, Sweden; 4 Department of OncologyHaukeland University Hospital, Bergen, Norway; 5 Department of Clinical ScienceUniversity of Bergen, Bergen, Norway; 6 Department of Hepatology and GastroenterologyAarhus University Hospital, Aarhus, Denmark; 7 Department of NeuroscienceUppsala University, Uppsala, Sweden; 8 Department of Medical SciencesUppsala University, Uppsala, Sweden; 9 Division of Cardiovascular MedicineDepartment of Medicine, Stanford University, San Francisco, California, USA; 10 Department of Surgical SciencesEndocrine Surgery, Uppsala University, Uppsala, Sweden

**Keywords:** familial cancer, cancer predisposition, small intestinal carcinoid, oxidative stress, DNA excision-repair pathway

## Abstract

The genetics behind predisposition to small intestinal neuroendocrine tumors (SI-NETs) is largely unknown, but there is growing awareness of a familial form of the disease. We aimed to identify germline mutations involved in the carcinogenesis of SI-NETs. The strategy included next-generation sequencing of exome- and/or whole-genome of blood DNA, and in selected cases, tumor DNA, from 24 patients from 15 families with the history of SI-NETs. We identified seven candidate mutations in six genes that were further studied using 215 sporadic SI-NET patients. The result was compared with the frequency of the candidate mutations in three control cohorts with a total of 35,688 subjects. A heterozygous variant causing an amino acid substitution p.(Gly396Asp) in the MutY DNA glycosylase gene (*MUTYH*) was significantly enriched in SI-NET patients (minor allele frequencies 0.013 and 0.003 for patients and controls respectively) and resulted in odds ratio of 5.09 (95% confidence interval 1.56–14.74; *P* value = 0.0038). We also found a statistically significant difference in age at diagnosis between familial and sporadic SI-NETs. *MUTYH* is involved in the protection of DNA from mutations caused by oxidative stress. The inactivation of this gene leads to specific increase of G:C- > T:A transversions in DNA sequence and has been shown to cause various cancers in humans and experimental animals. Our results suggest that p.(Gly396Asp) in *MUTYH*, and potentially other mutations in additional members of the same DNA excision-repair pathway (such as the *OGG1* gene) might be involved in driving the tumorigenesis leading to familial and sporadic SI-NETs.

## Introduction

Small intestinal neuroendocrine tumors (SI-NETs) are slowly growing, serotonin-producing malignancies and originate from enterochromaffin cells scattered throughout the mucosa of the small intestine (Kulke & Mayer 1999). The majority of patients have metastases already at diagnosis, but the 5-year survival is reported to be over 75%, despite the presence of distant metastases (Janson *et al*. 1997). Patients usually present with unspecific clinical symptoms such as abdominal pain or symptoms of the carcinoid syndrome; i.e. mainly diarrhea and/or flush (Janson *et al*. 2014). The incidence of SI-NETs is increasing during the past few decades, exemplified by the analysis of the SEER database (https://seer.cancer.gov/faststats/) and a recent publication from Norway, where the incidence of SI-NETs was 1.72 per 100,000 inhabitants and the median age at diagnosis was 64 years (Yao *et al*. 2008, Sandvik *et al*. 2016). However, the real frequency of the disease may very well be considerably higher. In a study based on autopsies from the southern part of Sweden, an incidence of >5 per 100,000 inhabitants was reported (Berge & Linell 1976). In this series, the majority of patients had clinically silent tumors; in fact, 90% were diagnosed post mortem. Thus, the true prevalence of these tumors in the general population is likely heavily underestimated, when the prevalence is based only on clinically diagnosed tumors.

During the past two decades, efforts have been made to characterize the genetics of SI-NETs. The vast majority of studies have focused on genetic events that can be identified in tumors. One of the most recurrent copy number variants (CNVs) is the loss of the entire or most of chromosome 18, occurring in about 70–80% of cases (Lollgen *et al*. 2001, Kulke *et al*. 2008, Cunningham *et al*. 2011). Consequently, it has been speculated that a tumor suppressor gene responsible for the development of SI-NETs might be present on this chromosome. The gene *TCEB3C* (elongin A3) was identified as a possible candidate (Edfeldt *et al*. 2014). Elongin A3 is one of a few so far characterized imprinted genes located on chromosome 18, and it is showing one copy deletion in a majority of tumors, with reduced elongin A3 gene expression. Other frequent aberrations involve gain of chromosomes 4, 5, 14 and 20, as well as loss of 9, 11q and 16q (Andersson *et al*. 2009). Frameshift and heterozygous mutations involving the *CDKN1B* gene, coding for the tumor suppressor p27, were recently described in approximately 8 and 14% of the analyzed tumors (Francis *et al*. 2013, Crona *et al*. 2015). Furthermore, a recent study using exome sequencing pinpointed somatic mutations in genes involved in different processes such as chromatin remodeling, DNA damage, apoptosis, RAS signaling and axon guidance. About 50% of the tumors had deleted or mutated *SMAD* genes, suggesting an involvement of the TGF-β pathway in tumor formation. Single-nucleotide variants were found in *MEN1*, *FGFR2*, *HOOK3*, *EZH2*, *MLF1*, *CARD11*, *VHL*, *NONO* and *SMAD1*. The amplifications of AKT1 or AKT2 were the most common alterations detected in the cases with an alteration of the PI3K/Akt/mTOR signaling pathway (Banck *et al*. 2013).

SI-NETs have long been considered and treated as sporadic conditions, and information about genetic risks in the germline is limited. However, a heritable component of the disease has been suggested, especially after observing that the relative risk for the offspring of parents having any cancer or specifically a neuroendocrine tumor is about 4.5–6.5 times higher than that in the rest of the population (Hemminki & Li 2001, Hiripi *et al*. 2009, Neklason *et al*. 2016). The relative risk of developing the disease having a sibling already affected with the same condition is estimated to be even higher (13.4-fold). We published a series of 10 families with SI-NETs and could show that deletion of chromosome 18 in tumors was less frequently found in these familial cases as compared to sporadic patients (Cunningham *et al*. 2011). Others have also reported families with SI-NETs (Wale *et al*. 1983, Kinova *et al*. 2001, Pal *et al*. 2001, Jarhult *et al*. 2010). Furthermore, a group at NIH presented a series of 33 families, having at least two SI-NET patients, and could identify a 4-bp deletion in the inositol polyphosphate multikinase gene (*IPMK*) segregating in one large family (Sei *et al*. 2015).

Unlike other endocrine tumor syndromes, such as multiple endocrine neoplasia type 1 and 2, which are inherited as autosomal dominant traits with well-defined genes causing these conditions, the possible genetic determinants of predisposition to SI-NETs remain poorly understood. A possible reason why the inherited component of SI-NETs has been underestimated may be a combination of late disease onset, unspecific symptomatology (abdominal pain and/or diarrhea as predominant symptoms), and that a large fraction of affected individuals never develop a clinically diagnosed tumor, resulting in skipped generations in family trees. Nevertheless, the genetic mechanism behind inherited SI-NETs is unlikely a single gene with autosomal dominant mode of inheritance and with high penetrance.

Considering the above mentioned premises, we applied a novel approach for analysis of SI-NET patients, based primarily on study of the constitutional DNA in familial and sporadic SI-NET patients, in an attempt to delineate genetic events that might predispose for this disease. The rationale and hypothesis for our study was that there is a subgroup of SI-NET patients with an inherited component behind disease development, since families with this disease have been reported (Wale *et al*. 1983, Kinova *et al*. 2001, Pal *et al*. 2001, Jarhult *et al*. 2010, Cunningham *et al*. 2011, Sei *et al*. 2015). Furthermore, we hypothesized that germline mutations (studied via analyses of blood DNA), that might be identified in familial subjects, should also be detectable in apparently sporadic patients with the disease.

## Materials and methods

### Familial SI-NET patients

**Figure 1:**
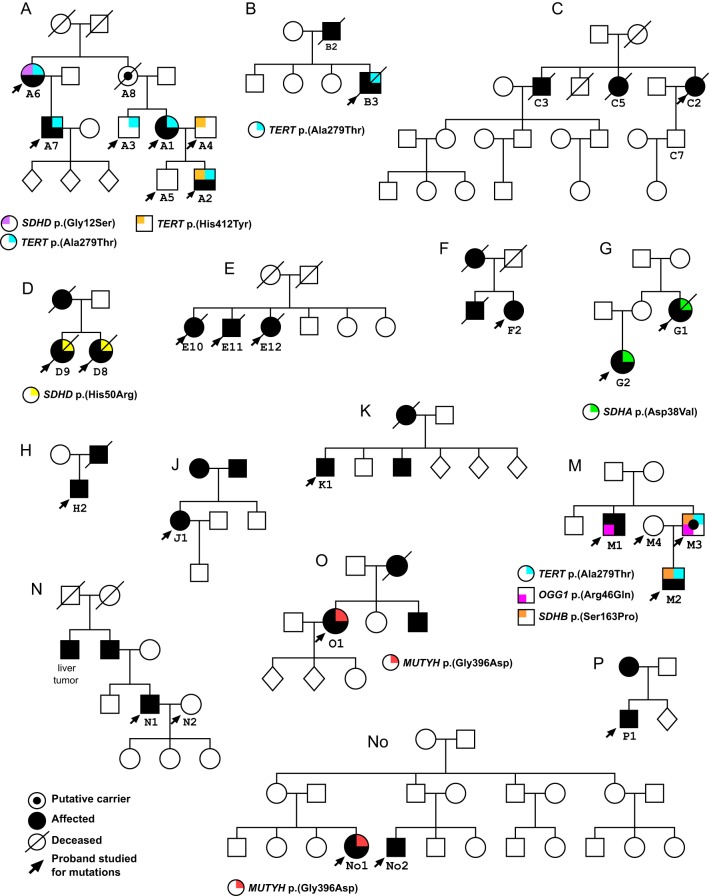
Pedigrees and germline variants detected in families with a history of SI-NETs. Clinically affected subjects are indicated by filled symbols. Females are designated by circles and males by squares, while individuals with unknown gender are depicted as diamonds. All affected and controls subjects studied for mutations are marked by arrows. Different variants that were considered as candidates for mutations contributing to disease development are depicted in different colors and explained in the legend under each pedigree. All these variants were present as a single allele in the germline of the tested individuals.

A total of 15 families with at least two documented individuals affected with SI-NETs were included in the study. All familial samples were collected at the Department of Endocrine Oncology at Uppsala University Hospital, Sweden, with the exception of two subjects from a Norwegian family ‘No’ (Fig. 1). The diagnosis and tumor classification were made according to international guidelines (Rindi *et al*. 2010, Jann *et al*. 2011). We collected clinical information about 26 patients affected with hereditary disease, with median age at disease onset of 57 years (range 34–68 years) and median survival of 83 months (Table 1). Blood DNA was also studied from three unaffected controls from families A, M and N (subjects A4, M4 and N2) and the healthy putative carrier M3 (Fig. 1). Clinical details, tissue tested and type of experiments performed for familial subjects are described in Table 2.

**Table 1:** Summary of clinical data comparing hereditary and sporadic patients.

**Patients**	**Hereditary** (*n* = 26)	**Sporadic** (*n* = 215)
WHO grade 1^a^	10	115
WHO grade 2^a^	8	45
Unknown tumor grade	8	55
TxNxM0^b^	5	61
TxNxM1^b^	20	147
Unknown tumor stage	1	7
Dead with disease	13	111
Alive with disease	11	96
No follow-up information	2	8
Median age at diagnosis	57 (34–68)	61 (23–90)
Median survival (months)	83 (40–348)	92.5 (2–348)

a Rindi G, Arnold R, Bosman FT. Nomenclature and classification of neuroendocrine neoplasms of the digestive system. In: Bosman FT, Carneiro F, Hruban RH, Theise ND *et al*., editors. WHO classification of tumors of the digestive system. Lyon: IARC, 2010; ^b^Tumors classified according to: Rindi G, Kloppel G, Alhman H *et al*. TNM staging of foregut (neuro)endocrine tumors: a consensus proposal including a grading system. Virchows Arch 2006, 449:395–401.

**Table 2:** Summary of clinical and experimental data in studied subjects from 15 families with small intestine neuroendocrine tumors (SI-NETs).

								**TERT Variants**	Variants in SDH genes			**Variants in excision-repair genes**		Experiments performed			
**Family no./ID**	**Subject ID**	**Gender**	**Affected** (Yes/No)	**Age at diagnosis**	**Survival** (years)^a^	**WHO**^b^	**Tumor stage**^c^	*TERT*	*SDHA*	*SDHB*	*SDHD*	*MUTYH*	*OGG1*	NGS of blood DNA^d^	NGS of tumor DNA^d^	Illumina SNP array on blood DNA^e^	Illumina SNP array on tumor DNA^e^
1/A	A1	F	Y	45	27.3 (AWD)	1	TxN1M1	p.(Ala279Thr)						WGS (35×) WES (78×)	WES (25×)	610Q 2.5 M Omni	610Q (PT)
	A2	M	Y	34	13.5 (AWD)	1	TxN1M1	p.(Ala279Thr) p.(His412Tyr)						WGS (35×) WES (89×)	–	2.5 M Omni	–
	A3	M	N	–	–	–	–	p.(Ala279Thr)						–	–	2.5 M Omni	–
	A4	M	N	–	–	–	–	p.(His412Tyr)						WES (146×)	–	2.5 M Omni	–
	A5	M	N	–	–	–	–							–	–	2.5 M Omni	–
	A6	F	Y	35	n.a.	n.a.	n.a.	p.(Ala279Thr)			p.(Gly12Ser)			WES (164×)	–	2.5 M Omni	–
	A7	M	Y	42	6.2 (AWD)	2	TxN1M1	p.(Ala279Thr)						WGS (33×) WES (87×)	–	2.5 M Omni	–
2/B	B1	F	N	–	–	–	–							–	–	2.5 M Omni	
	B2	M	Y	62	15**^+^**	n.a.	TxN1M1							–	–	–	610Q (M)
	B3	M	Y	42	5.8**^+^**	2	TxN1M1	p.(Ala279Thr)						WES (89×)	WES (27×)	610Q 2.5 M Omni	2.5 M Omni (PT)
3/C	C2	F	Y	68	5.2**^+^**	2	TxN1M1							WES (78×)	–	–	–
4/D	D8	F	Y	63	4.3**^+^**	n.a.	TxN1M1				p.(His50Arg)			WGS (32×)	–	610Q 2.5 M Omni	–
	D9	F	Y	59	11.7**^+^**	1	TxN1M1				p.(His50Arg)			WGS (27×)	–	610Q 2.5 M Omni	–
5/E	E10	F	Y	62	6.3**^+^**	n.a.	TxN1M0							WES (111×)	–	2.5 M Omni	–
	E11	M	Y	67	19.4**^+^**	n.a.	TxN1M0							WES (129×)	–	2.5 M Omni	–
	E12	F	Y	62	5.4**^+^**	1	TxN1M1							WES (188×)	–	2.5 M Omni	–
6/F	F1	M	Y	38	29**^+^**	n.a.	TxN1M1							–	–	–	
	F2	F	Y	66	6.3 (AWD)	1	TxN1M1							WES (89×)	WES (28×)	610Q 2.5 M Omni	610Q (M) 610Q (PT) 2.5 M Omni (PT)
7/G	G1	F	Y	68	6.4**^+^**	1	TxN1M1		p.(Asp38Val)					WGS (33×) WES (87×)	–	610Q 2.5 M Omni	610Q (M)
	G2	F	Y	50	n.a.	n.a.	TxN1M0		p.(Asp38Val)					WGS (34×)	–	610Q 2.5 M Omni	610Q (M)
8/H	H2	M	Y	52	8**^+^**	1	TxN1M1							WES (81×)	WES (35×)	610Q 2.5 M Omni	610Q (M)
9/J	J1	F	Y	40	12 (AWD)	1	TxN1M1							WES (139×)	–	2.5 M Omni	–
10/K	K1	M	Y	60	6.9 (AWD)	2	TxN1M1							WES (200×)	–	2.5 M Omni	–
11/M	M1	M	Y	66	8.8**^+^**	2	TxN1M1						p.(Arg46Gln)	WES (74×)	–	2.5 M Omni	–
	M2	M	Y	43	4 (AWD)	2	TxN1M1	p.(Ala279Thr)		p.(Ser163Pro)				WES (88×)	–	2.5 M Omni	–
	M3	M	N	–	–	–	–	p.(Ala279Thr)		p.(Ser163Pro)			p.(Arg46Gln)	WES (91×)	–	–	–
	M4	F	N	–	–	–	–							WES (88×)	–	–	–
12/N	N1	M	Y	63	2.8 (AWD)	1	TxN1M0							WES (91×)	–		
	N2	F	N	–	–	–	–							WES (77×)	–		
13/No	No1	F	Y	57	10.8 (AWD)	1	T3N2M1					p.(Gly396Asp)		WES (86×)	–	–	–
	No2	M	Y	49	3.8 (AWD)	2	T3N0M1							WES (78×)	–	–	–
14/O	O1	F	Y	57	3.4 (AWD)	2	TxN1M0					p.(Gly396Asp)		WES (80×)	–	2.5 M Omni	
15/P	P1	M	Y	39	20+	n.a.	TxN1M1							WES (82×)	–	–	–

a AWD, alive with disease; ^+^deceased; ^b^grade of tumor according to Rindi G, Arnold R, Bosman FT. Nomenclature and classification of neuroendocrine neoplasms of the digestive system. In: Bosman FT, Carneiro F, Hruban RH, Theise ND *et al*., editors. WHO classification of tumors of the digestive system. Lyon: IARC, 2010; ^c^tumors classified according to: Rindi G, Kloppel G, Alhman H, Caplin M, Couvelard A *et al*. TNM staging of foregut (neuro)endocrine tumors: a consensus proposal including a grading system. Virchows Arch 2006, 449:395–401; ^d^abbreviations: WGS, whole-genome sequencing. WES, whole-exome sequencing. The number in parentheses denotes the read depth of the NGS experiments. The coverage was calculated for WES data by using the software GATK (DeptOfCoverage) on exome target regions, while for whole-genome experiments the overall coverage was calculated by extracting the total base coverage from BAM alignments with Samtools (depth) and then dividing this number by the human genome size (3.1 billion bases); ^e^DNA extracted from blood and tumors (in parenthesis: PT-primary tumor; M-metastasis) was genotyped on two platforms: Illumina Human610-Quad BeadChip (610Q) and Illumina Omni 2.5 BeadChip (2.5 M Omni).

n.a., data not available.

### Sporadic SI-NET patients

296 samples from 215 unrelated sporadic SI-NET patients were collected at the Departments of Endocrine Oncology and Endocrine Surgery at Uppsala University Hospital, Sweden. The clinical details, type and number of samples tested for each of them are described in Supplementary Table 1 (see section on supplementary data given at the end of this article). Tumor classification and tumor staging were obtained in the same fashion as for familial subjects. Median age at diagnosis was 61 years (range 23–90 years) and median survival for sporadic patients was 92.5 months (Table 1). DNA from blood, normal tissue (NT) or tumor, either primary tumor (PT) or metastasis (M), was available for analysis from sporadic patients. All tumor samples were macro-dissected to contain at least 80% tumor cells. The research protocol used in the current study was approved by the local research ethics committee in Uppsala, Sweden, and all included patients (both familial and sporadic) signed the informed consent.

### Control populations

The vast majority of familial and sporadic cases included in our collection of patients have a Scandinavian background (mostly born and living in Sweden). For assessment of allele frequencies in the general population, we used three different studies: (i) the Exac Aggregation Consortium, European fraction, consisting of 33,370 individuals (Lek *et al*. 2016) and (ii) 1000 Genomes project (http://www.internationalgenome.org/). This cohort was consisting of 503 individuals of European ancestry (Genomes Project Consortium *et al*. 2015); (iii) the Swedish EpiHealth study (Epidemiology for Health, https://www.epihealth.se/), which started in 2011 and is a population-based cohort from Uppsala for analysis of gene–lifestyle interactions (Lind *et al*. 2013). We decided to exclude the Finnish component of Exac and 1000 Genomes from our statistics because of the peculiarity of its genetics. Finns have been shown to be a bottlenecked population, descending from a limited number of families and therefore differing from the rest of Europeans (Lek *et al*. 2016). The frequency of variants in Exac and 1000 Genomes projects is known and registered in these respective databases, while these numbers had to be calculated from the genotyping results in the case of the EpiHealth controls. In order to calculate the final frequency of the candidate variants detected in SI-NET patients, we used the EpiHealth cohort, which has a very similar ancestry. The age range of EpiHealth participants is 45–75 years and the phenotypic scope of EpiHealth study is broad, including cancer. A random selection of 2500 participants from EpiHealth cohort was genotyped using Illumina beadchip, allowing analysis of all seven DNA variants. This chip was designed to contain ~538 K probes covering mostly exons and other disease-related regions of interest. Importantly, in EpiHealth cohort we were also able to exclude all subjects that were affected by cancer at the entry to the study and 2318 individuals (with a similar genetic background to the familial and sporadic SI-NET patients) were included in our analysis.

### Next-generation sequencing analysis

We applied a combination of next-generation highly parallel sequencing (NGS) of exome and/or whole genome. NGS experiments were performed on blood from 25 subjects (24 affected and one putative healthy carrier; M3 from family M) with familial history of the disease and 3 control subjects from 3 distinct families (A, M and N). Seven whole-genome sequencing (WGS) experiments were performed on blood DNA from subjects from families A, D and G with a mean depth of coverage of ~30×. The remaining samples were exome sequenced either at the National Genomics Infrastructure, Stockholm, Sweden, with a mean coverage depth of ~90× or at the Genome Center in Vancouver, Canada, with a mean depth of coverage of ~120×. The exomes of additional four tumors from subjects A1, B3, F2 and H2 were sequenced at a lower coverage (~25×), allowing to compare the genetic profiles of blood and paired tumor DNA for these patients (Table 2). Data pre-processing, variant calling and refinement were accomplished by following the Broad Institute’s GATK (https://software.broadinstitute.org/gatk/) Best Practices workflow (Supplementary Fig. 1). Raw sequence data from each WES and WGS experiment were aligned independently to the human reference NCBI Build 37 using Burrows-Wheeler Aligner (BWA), version 0.7.5 (http://bio-bwa.sourceforge.net/). Reads were de-duplicated using Picard-Tools, version 1.94 (https://broadinstitute.github.io/picard/).

GATK, version 3.4 was used for SNPs and INDELs realignment and base quality recalibration. The same software was adopted for raw variant calling (HaplotypeCaller) and SNPs and INDELs hard filtering. We then used ANNOVAR (http://annovar.openbioinformatics.org/en/latest/) for annotating and filtering called variants (Wang *et al*. 2010). We subsequently filtered out single nucleotide variants (SNVs), insertions and indels with frequency higher than 5% in the Exome Aggregation Consortium (Exac, version 0.3; http://exac.broadinstitute.org/). Exac was chosen as one of references because, although containing a portion of data derived from cancer projects such as The Cancer Genome Atlas (TCGA), it is the biggest NGS dataset available to date, containing data from more than 60,000 unrelated individuals (Lek *et al*. 2016). All steps applied to the data in order to extract a list of potentially damaging and recurring across families variants, are described in Supplementary Fig. 1.

### SNP array analysis of familial cases and EpiHealth controls

We performed copy number variant analysis from SNP genotyping experiments on DNA from control and tumor tissues from patients with familial history of SI-NETs. Genotyping experiments were performed on two distinct Illumina platforms: the Human610-Quad and the 2.5 M Omni Beadchips, which have a density of 610 K and 2.5 M probes respectively. Experiments executed on the 610 K platform are described in the study by Cunningham and coworkers (Cunningham *et al*. 2011). The advantage of the SNP array technology is the possibility to analyze two tracks at the same time: log R ratio (LRR), which is indicative of the DNA copies for each probe, and the B allele frequency (BAF), which instead illustrates the relative ratio between the alleles. DNA samples from familial cases were genotyped at the SNP&SEQ Technology Platform in Uppsala, according to the instructions provided by the manufacturer. A SNP call rate >98% and a LogRdev <0.2 were used to determine the experiments passing quality criteria. Illumina raw files were exported using the Genome Studio Nexus Copy Number Plugin and analyzed using the Nexus Copy Number software, version 7.5 (Biodiscovery, CA, USA). In order to call structural variants from genomic profiles, we applied the SNP-FASST2 Nexus segmentation algorithm, which is based on Hidden Markov Model (HMM) approach and combines data from both LRR and BAF for segmentation (Table 2). We also used SNP array data from the EpiHealth cohort to test the frequency of candidate alleles in this control population. For these, we applied the same filtering criteria used for the familial patients.

### Sanger sequencing validation of familial germline variants in sporadic subjects

After having identified candidate variants from WGS and WES experiments on familial SI-NETs cases, we designed validation primers for seven loci using PrimerZ (Tsai *et al*. 2007), using the option ‘Input SNPs or Positions’ with default settings except for the product size ranges that was restricted to 100–400 base pairs (http://genepipe.ngc.sinica.edu.tw/primerz/). Primer sequences and PCR conditions are described in Supplementary Table 2. PCR bands were purified from agarose gel and Sanger sequenced on a ABI3730XL DNA Analyzer (Thermo Fisher Scientific) at The Uppsala Genome Center, Science For Life Laboratory in Uppsala, Sweden. The same seven primer pairs designed for the familial cases were used to investigate the presence of the same variants in the 296 sporadic samples. In this case, after the PCR amplification step, samples were directly treated with the ExoSAP-IT (Thermo Fisher Scientific) prior to performing Sanger sequencing at the same facility.

### Immunohistochemistry

Paraffin-embedded tumor tissue specimens from SI-NET patients with and without verified mutations in *MUTYH*, *OGG1 TERT*, *SDHB* and *SDHD* were cut into approximately 4-µm thick sections and attached to positively charged glass slides (Superfrost Plus, Menzel Gläser, Braunschweig, Germany). Before immunostaining, the sections were treated in a pressure cooker reaching maximum temperature of 121°C using citrate buffer pH 6.0, as retrieval solution. The sections were incubated with the primary polyclonal antibody anti-MUTYH (1:10, HPA008732, Atlas Antibodies, Bromma, Sweden) or anti-OGG1 (1:500, PA1-16505, Thermo Fisher Scientific) at room temperature for one hour. A polymer-detection system was used (EnVision Plus-HRP, Dako) according to manufacturer’s instructions. Diaminobenzidine was used as a chromogen for five minutes. Tissue sections from normal liver and normal tonsil were used as positive controls for MUTYH and OGG1 respectively (not shown), and omission of the primary antibody was used as a negative control.

## Results

### Characterization of candidate mutations predisposing to SI-NETs in families

The analysis of 15 families with SI-NETs was the starting point for our investigation (Fig. 1). All but one family were Swedish and the exception is family ‘No’, which was collected in Norway. Overall clinical summary and details for 26 familial SI-NET patients that were studied molecularly are shown in Tables 1 and 2. Only one of the families had four affected subjects (family A). This was also the only kindred involving more than two generations of patients affected with SI-NETs. All other families had two to three affected individuals, ascertained across two generations only. Blood-derived DNA was sequenced for 24 familial SI-NET patients, as well as selected healthy members of families (Table 2). As shown in Table 1, comparison of median age at diagnosis between familial and sporadic SI-NET patients suggested a difference between these two groups (Table 1). We found that age at diagnosis was significantly lower in familial SI-NET patients compared to sporadic SI-NET patients (Mann–Whitney *U* test; *W* = 1789.5, *P* = 0.0054). As shown in Table 1, the median age at diagnosis among 26 familial cases and 207 sporadic cases was 57 and 61 years of age respectively.

**Figure 2:**
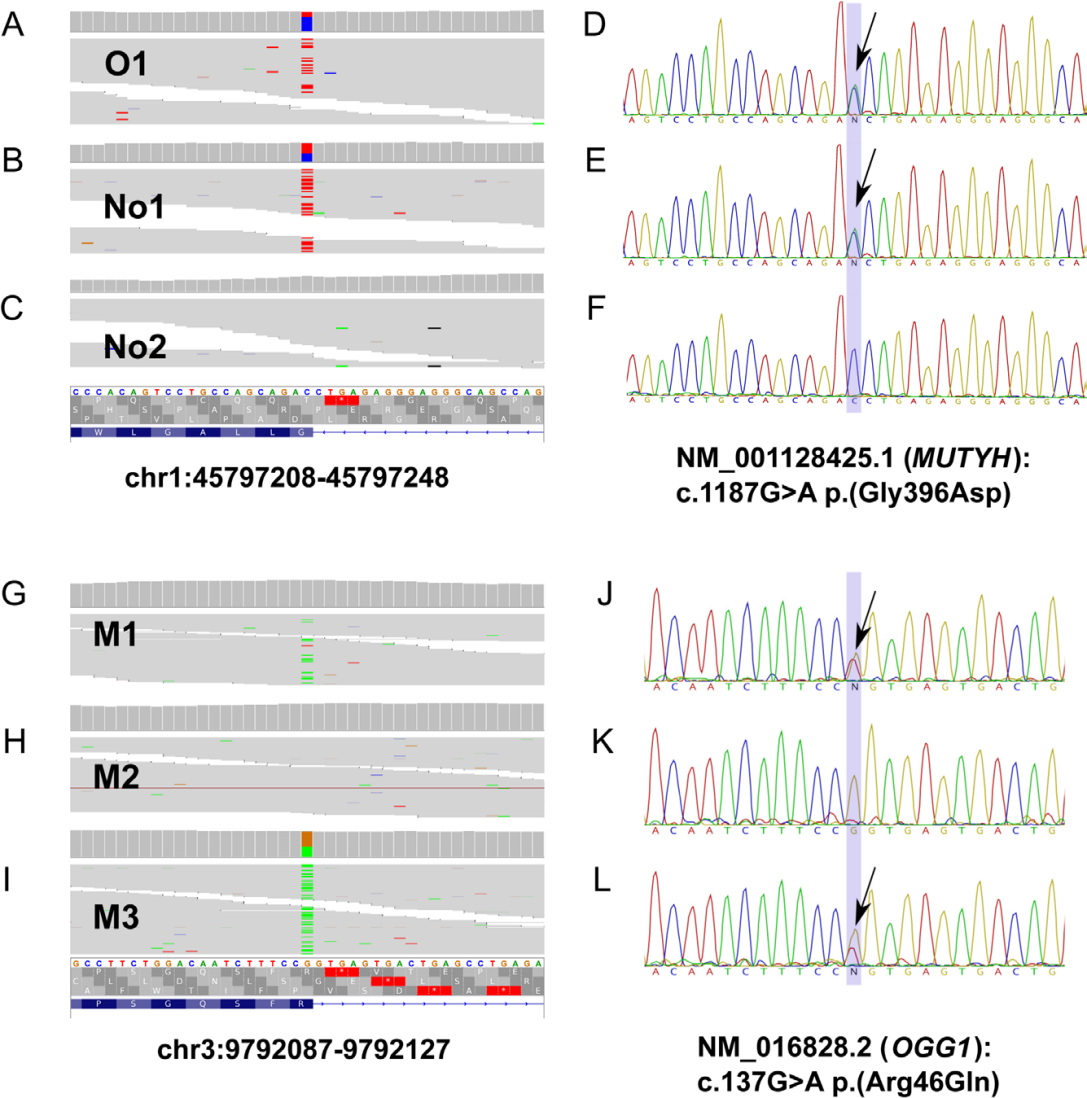
Identification and validation of genetic variants in *MUTYH* and *OGG1* genes in the germline of three families with SI-NETs. Whole-exome sequencing results (panels A, B, C and G, H, I) with corresponding Sanger sequencing validations (panels D, E, F and J, K, L) of heterozygous variants *MUTYH* p.(Gly396Asp) and *OGG1* p.(Arg46Gln) in the germline of subjects with familial history of SI-NETs. NGS data are presented using the Integrative Genomics Viewer – IGV software, Broad Institute. Variant *MUTYH* p.(Gly396Asp) was initially detected in two families with history of SI-NETs; families O and No. The sequenced subject from family O, (subject O1 (panels A and D)), is a carrier of this variant. The same substitution was also detected in one of the two sequenced subjects of family No, i.e. No1 (panels B and E). The variant was not identified in the other affected subject of the same family, No2 (panels C and F). Panels G to L illustrate the presence of variant *OGG1* p.(Arg46Gln) in two subjects of family M (M1 (panels G and J) and M3 (panels I and L)). Subject M1 was diagnosed with the disease at the age of 66 years, while his younger brother M3 is considered to be a healthy carrier, since his son M2 (panels H and K) was diagnosed with SI-NET at the age of 43 years. M2 did not show any *OGG1* mutation, but he is a carrier of heterozygous alleles *TERT* p.(Ala279Thr) and *SDHB* p.(Ser163Pro) (Fig. 1, Tables 2 and 3).

In the analysis of familial patients, we applied a combination of next-generation highly parallel sequencing (NGS) of exome- and/or whole-genome and targeted Sanger sequencing as well as SNP-beadchip analysis of blood and/or tumor DNA. For analysis of NGS data, we used a pipeline of stepwise filtering of results from sequencing of the genomes and exomes. Table 3 and Supplementary Fig. 1 describe in detail the filtering steps applied in the analysis of NGS data. After the initial steps of alignment, variant calling and refinement, we compiled a list of candidate variants for each family. We populated these lists with variants present in one (for families with only one affected subjects sequenced) or more cancer patients, prioritizing those reported as pathogenic (specifically cancer causing) in ClinVar database (https://www.ncbi.nlm.nih.gov/clinvar/) or predicted to be damaging by at least one of the three functional prediction algorithms: SIFT, Polyphen and MutationTaster (Kumar *et al*. 2009, Schwarz *et al*. 2010, Adzhubei *et al*. 2013). Data from different families were integrated to identify candidates recurring across different families, protein members of the same complex or interacting in the same biological pathway. To further exclude sequencing errors and before the final validation by Sanger sequencing, we manually inspected alignments containing candidate variants using the Integrative Genomics Viewer software (http://software.broadinstitute.org/software/igv/) (Fig. 2).

**Table 3:** Association between seven heterozygous missense variants in six candidate genes and small intestinal neuroendocrine tumors (SI-NETs).

				Variant effect prediction			**Minor allele frequency (MAF) in control cohorts**			**MAF in germline of SI-NET patients compared to the EpiHealth** (two-tailed Fisher’s exact test)^e^				**MAF in germline and/or tumor of SI-NET patients compared to the EpiHealth** (two-tailed Fisher’s exact test)^e^			
**rsID**	**Gene**	**cDNA**	**Amino acid residue change**	SIFT	Polyphen	Mut.Taster	1000G (EUR) MAF^a^	Exac 0.3 (EUR) MAF^b^	EpiHealth controls MAF^c,d^	SI-NETs MAF (germline)^c^	OR	95% CI	*P*-Value	SI-NETs MAF (cancer+germline)^c^	OR	95% CI	*P*-Value
rs61748181	*TERT*	C/T	p.(Ala279Thr)	Tolerated	Probably damaging	Disease causing	0.036	0.05	0.036 (167/4636)	0.028 (9/316)	0.76	0.35–1.55	0.64	0.027 (12/444)	0.74	0.37–1.35	0.42
rs34635677	*SDHA*	A/T	p.(Asp38Val)	Tolerated	Benign	Polymorphism	0.032	0.044	0.034 (157/4636)	0.032 (10/316)	0.93	0.43–1.78	1	0.035 (16/458)	1	0.57–1.75	0.89
rs33927012	*SDHB*	T/C	p.(Ser163Pro)	Tolerated	Benign	Disease causing	0.017	0.015	0.02 (96/4636)	0.013 (4/316)	0.6	0.16–1.62	0.41	0.011 (5/460)	0.52	0.16–1.26	0.22
rs11214077	*SDHD*	A/G	p.(His50Arg)	Tolerated	Probably damaging	Disease causing	0.017	0.01	0.01 (46/4636)	0.013 (4/318)	1.27	0.33–3.51	0.56	0.011 (5/460)	1.1	0.34–2.77	0.8
rs34677591	*SDHD*	G/A	p.(Gly12Ser)	Tolerated	Benign	Disease causing	0.01	0.01	0.008 (36/4636)	0.009 (3/318)	1.22	0.24–3.88	0.74	0.007 (3/460)	0.84	0.16–2.67	1
rs36053993	*MUTYH*	G/A	p.(Gly396Asp)	Damaging	Probably damaging	Disease causing	0.009	0.004	0.003 (12/4636)	0.016 (5/316)	6.19	1.7–19.02	0.0034	0.013 (6/460)	5.09	1.56–14.74	0.0038
rs104893751	OGG1	G/A	p.(Arg46Gln)	Damaging	Probably damaging	Disease causing	0.002	0.003	0.005 (22/4636)	0.01 (3/314)	2.02	0.39–6.79	0.2	0.009 (4/460)	1.84	0.46–5.45	0.29

a Allele frequencies in this column are based on the European (Non-Finnish) fraction of the 1000 Genomes project, consisting of 503 subjects; ^b^allele frequencies in this column are based on the European (Non-Finnish) fraction of the Exac Aggregation Project, consisting of 33,370 subjects; ^c^minor allele frequencies (MAFs) were calculated for control and case cohorts by dividing the the number of minor alleles detected by all possible alleles in the population tested. These numbers are reported in parenthesis under the MAFs. The numbers for patients affected with the disease include 15 families, counted as one subject per family affected with SI-NET. Thus, the familial cases add a maximum of 30 alleles to the calculations of MAF in the cohort of patients affected with SI-NETs; ^d^the control cohort used for calculations was the Swedish EpiHealth study; composed of 2318 individuals with the same genetic background as the affected subjects; ^e^variants were tested for statistical significance by comparing their prevalence in germline or in germline and/or tumor of affected subjects to their frequency in the EpiHealth. Subjects diagnosed with any typer of cancer were excluded from the EpiHealth cohort before analysis. A two-tailed Fisher’s exact test was used on 2 × 2 contingency tables containing allele MAFs of cases and controls to obtain odds ratios (ORs), 95% confidence intervals (CIs) and *P* values for each comparison. No correction for multiple testing has been done, since only one test was significant (p.(Gly396Asp) in *MUTYH* and the *P* value was very small.

The heterozygous missense variant in the telomerase reverse transcriptase gene (*TERT*) p.(Ala279Thr) was the most common DNA sequence variant identified among the 24 familial SI-NET patients and was observed in six patients from three families (subjects A1, A2, A6, A7, B3 and M2) (Fig. 1, Table 2 and Supplementary Fig. 2). Prediction of the phenotypic effect of this variant was discordant in SIFT, Polyphen and MutationTaster; reported as ‘tolerated’, ‘probably damaging’, and ‘disease causing’ respectively. Furthermore, one unaffected older brother of subject A1 (subject A3, clinically healthy and currently 75 years of age) also showed this DNA sequence variant. A similar situation was encountered in family M, where the unaffected subject M3 had the variant *TERT* p.(Ala279Thr). In addition, in two members of family A, an additional heterozygous variant of the *TERT* gene p.(His412Tyr) was uncovered. It was present in A4 (married to affected subject A1) and he passed on this allele to his affected son A2, who is therefore a compound heterozygote for two *TERT* gene variants; p.(Ala279Thr) and p.(His412Tyr). SIFT, Polyphen and MutationTaster predicted this variant in a similar way as the former one (tolerated, probably damaging and disease causing respectively) (Table 3). Both Ala279Thr and His412Tyr variants are described as pathogenic in ClinVar and were reported in patients affected with bone marrow failure, aplastic anemia and dyskeratosis congenita, a telomere-related disorder (Vulliamy *et al*. 2005, Yamaguchi *et al*. 2005). This variant has been furthermore described in esophageal carcinomas, both in heterozygous and homozygous states. In comparison with normal ones, cells expressing the *TERT* A279T variant were shown to have shorter telomeres and impaired canonical and non-canonical telomerase functions (Zhang *et al*. 2014).

NGS analysis of the whole-exome and whole-genome data on the familial cases also revealed four likely polymorphisms in three genes (*SDHA*, *SDHB* and *SDHD*) encoding different subunits of the mitochondrial succinate dehydrogenase complex (Fig. 1, Table 2 and Supplementary Figs 3, 4 and 5). These were found in blood DNA of seven familial subjects (out of 24; 29%) from four families (A, D, G and M). These four variants showed conflicting results regarding the effect prediction (Table 3). Notably, we did not detect any nucleotide variants affecting the subunit C of the succinate dehydrogenase complex (*SDHC*). The *SDHD* His50Arg and Gly12Ser variants have previously described neuroendocrine tumors such as pheochromocytomas and paragangliomas, as well as midgut carcinoids and Merkel cell carcinomas (Cascon *et al*. 2002*b*, Kytola *et al*. 2002, Perren *et al*. 2002, Ni *et al*. 2008). The pathogenicity of *SDHB* Ser163Pro according to ClinVar is controversial, but this allele has previously been detected in familial cases of pheochromocytoma and paraganglioma (Cascon *et al*. 2002*a*). A polymorphism affecting the A subunit of the complex, *SDHA* Asp38Val, detected in two affected individuals of family G, G1 and G2, has been previously described in gastrointestinal stromal tumors (Italiano *et al*. 2012). The above studies describe homozygous inactivation as the most plausible mechanism of tumorigenesis for these variants of the mitochondrial SDHC. We have, however, not observed biallelic mutations when the variants are detected in the germline of patients affected with familial SI-NETs.

Furthermore, a heterozygous variant causing an amino acid substitution p.(Gly396Asp) in the MutY DNA glycosylase gene (*MUTYH*) was observed in two SI-NET patients from different families (subjects O1 and No1) (Fig. 1), and this DNA sequence change was considered damaging by all three prediction algorithms (Table 3). Germline biallelic variants of *MUTYH* (including Gly396Asp) have been previously described in patients affected with multiple colorectal adenomas and adenomatous polyposis, as well as pancreatic NETs (Al-Tassan *et al*. 2002, Sampson *et al*. 2003, Sieber *et al*. 2003, Vogt *et al*. 2009, Scarpa *et al*. 2017). Finally, in family M (subject M1), we also observed a variant in the 8-oxoguanine DNA glycosylase gene (*OGG1*) p.(Arg46Gln), which was predicted as damaging by all three methods, and this gene was therefore considered a candidate for further analysis. This variant was also present in subject M3 (putative carrier, Fig. 1), who is currently 73 years and clinically healthy. A study of human kidney carcinomas has suggested that this variant might be a risk allele (Audebert *et al*. 2000). It is also noteworthy that the *OGG1* gene encodes a protein that is functionally closely related to *MUTYH*; both proteins are involved in the protection of DNA from mutations caused by oxidative damage (see below, Discussion section).

In summary, for the familial SI-NET patients, we analyzed 15 small families and identified seven heterozygous missense variants affecting six genes, which could be further tested in sporadic SI-NET patients (see below). All identified variants were reported as involved in cancer in ClinVar and pinpointed as possible pathogenic by our unbiased filtering pipeline of NGS data; thus, both approaches converged on the same candidate mutations. Family A and M are the two families that showed the largest load of uncovered variants. In eight out of our 15 families, we were unable to identify any DNA sequence variants that could be considered candidates for further analysis (Fig. 1). All the missense variants described above were present as a single allele in the blood DNA of the tested individuals, which is presumed to represent the germline variation. The overall picture of observed variation suggests heterogeneity of mechanisms involved in the development of familial form of SI-NETs. The studied families are not suitable for linkage studies and should be considered as familial aggregations, instead of clear-cut kindreds with Mendelian autosomal dominant inheritance pattern and with high penetrance, which is typical for inherited cancer syndromes. It should also be mentioned that we tested, with negative results (details not shown), our families for mutations in the inositol polyphosphate multikinase gene (*IPMK*), which has been reported in one large family with SI-NETs (Sei *et al*. 2015).

### Sporadic and familial patients reveal that mutations in *MUTYH* are associated with SI-NETs

**Figure 3:**
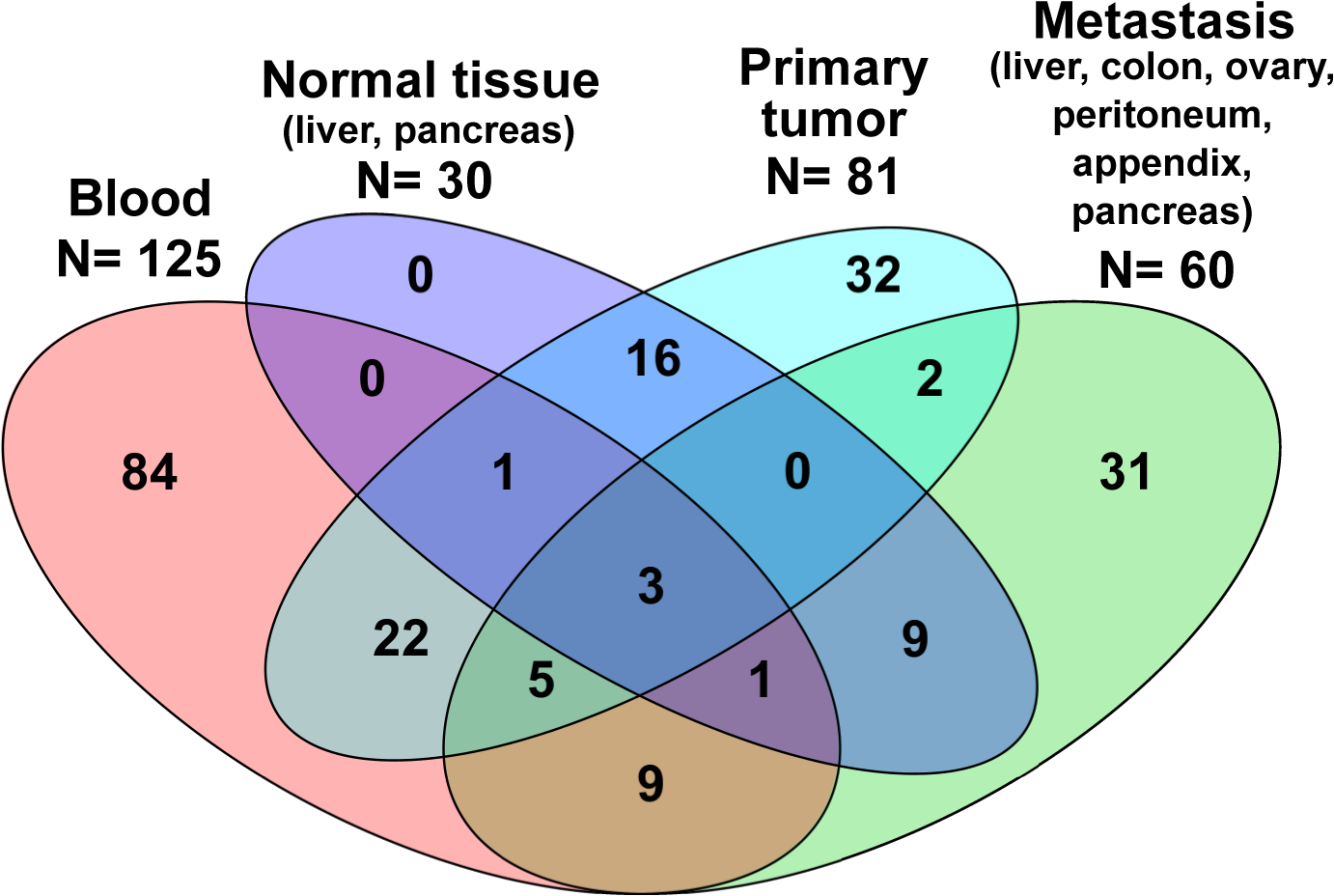
Venn diagram showing the different tissues analyzed for the sporadic SI-NETs. DNA extracted from a total of 296 sample tissues from 215 unrelated subjects was screened for the seven candidate variants. Samples were available from tumors (PT and/or metastases) and cancer-free tissues (blood and/or NT from liver). Clinical details, type and number of samples tested for each affected individual are described in Supplementary Table 1. Samples for multiple tissues (two to four) were available for 68 sporadic patients while 147 of these had only one specimen represented in our collection. All subjects with a NT available for our analyses (*n* = 30) were also sampled for another tissue, either blood or tumor/metastasis.

All sporadic patients were treated and samples from them were collected at Uppsala University Hospital, Sweden. Samples obtained from 215 unrelated subjects affected with SI-NETs were screened for the seven candidate variants and the results are summarized in Table 3. DNA was extracted from a total of 296 samples from SI-NET patients, regarded to the best of our knowledge as sporadic cases and used for mutation analysis using Sanger sequencing. Samples were available from tumors (PT and/or metastases) and/or cancer free-tissues (blood and/or NT from liver). Figure 3 shows a Venn diagram summarizing the numbers and types of samples studied for sporadic patients. Samples for multiple tissues (two to four) were available for 68 sporadic patients, while 147 of patients were represented by one specimen only. It was possible to test constitutional DNA (from blood tissue or NT) against paired tumor (PT and/or metastasis) for 60 patients. We had the opportunity of studying DNA from the triad of samples (germline/tumor/metastasis) for nine subjects.

For assessment of allele frequencies in the general population, we used three different studies, as described (see ‘Materials and methods’ section). A comparison of allele frequencies for the seven variants showed that the heterozygous variant causing amino acid substitution p.(Gly396Asp) in *MUTYH* was significantly enriched among the patients affected with SI-NETs, compared to all the control cohorts. The minor allele frequencies (MAFs) for the *MUTYH* p.(Gly396Asp) variant were: 0.009, 0.004 and 0.003 for the Exac-, 1000 Genomes-, and EpiHealth cohort respectively. The overall minor allele frequency of this variant in our collection of affected subjects (sporadic and familial subjects) was 2–5 times higher (MAF = 0.016) when considering subjects harboring the variant in the germline. A similar situation was observed when studying variants found in any tissue (germline plus tumor samples) resulting in MAF = 0.013; 1.4–4 times higher than that in controls. To calculate these numbers, we took into account only one subject from each family with history of SI-NETs, specifically the oldest diagnosed with the disease. Calculations of odds ratios (Fisher’s exact test) for being affected with SI-NETs and having SNP causing amino acid substitution p.(Gly396Asp) in *MUTYH* were 6.19 (95% confidence interval (CI): 1.7–19.02, *P* value = 0.0034) and 5.09 (95% CI: 1.56–14.74, *P* value = 0.0038) (Table 3). The former calculations involved only cases studied using the germline DNA of patients with SI-NET and the latter was calculated also including data from tumors, in addition to the variation in the germline respectively. The 95% CIs for the odds ratios are wide, largely because of the low MAFs in controls, suggesting that future studies of p.(Gly396Asp) in *MUTYH* in SI-NET patients are necessary using a larger cohort of patients. The monoallelic mutation *MUTYH* p.(Gly396Asp) was also uncovered in two samples of tumor DNA; i.e. has not been eliminated by genomic rearrangements arising in the tumors. However, due to the unavailability of tumor specimens from the relevant patients, we have not been able to carefully study tumors from all patients for deletions on 1p (where *MUTYH* is located), with an aim to uncover the mechanism of inactivation of the other allele of the *MUTYH* gene. All other studied variants were either not enriched among studied patients vs controls or the differences were not statistically significant. The *OGG1* mutation p.(Arg46Gln) was enriched in SI-NETs about two times, compared to EpiHealth, but our patient cohort would need to be considerably larger in order to reach the statistical significance, suggesting that future studies of *OGG1* p.(Arg46Gln) candidate mutation in SI-NET patients are necessary. As for the mutation in *MUTYH* gene, also this variant in *OGG1* was present in a single copy in all studied subjects, including one metastasis and one PT from apparently sporadic patients.

### Analysis of protein expression in tumors from familial patients

We have also performed immunohistochemical analysis of the protein expression in tumors, for candidate genes that were studied for mutations in both familial and sporadic cohorts. Tumor samples (PT or liver metastases) from seven patients with the familial form of SI-NET were studied. All patients had at least one germline mutation, five had a *TERT* mutation, two had *MUTYH* mutations and *OGG1*, *SDHB* and *SDHD* were found in one patient each. Of the seven patients, one had three mutations (*TERT*, *MUTYH* and *OGG1*) and one had two (*TERT* and *SDHB*) (Table 2). All studied tumors showed immunoreactivity for antibodies used. In Supplementary Fig. 7, immunohistochemical analysis of MUTYH and OGG1 is shown. There was no difference in the staining pattern or intensity of the staining between tumors from patients with or without a germline mutation in *OGG1* or *MUTYH*. This indicates that the expression of the protein in tumors was not affected by the monoallelic mutations in the *MUTYH* or *OGG1* genes. A similar pattern has been observed in tumors from familial patients carrying other observed gene mutations (compared to controls without the mutation), detected with antibodies against TERT, SDHB and SDHD (results not shown). These results for the *SDHB* and *SDHD* genes may suggest that these mutations do not have an effect on the expression of proteins of the mitochondrial complex II, speaking against their possible role in this disease. This is in contrast to observations in pheochromocytomas and paragangliomas, where it has been shown that pathogenic variants of these genes, present in tumors in two copies, lead to loss of expression with subsequent impaired enzymatic activity (van Nederveen *et al*. 2009). The fact that normal and tumor tissues showed a similar expression of *TERT*, a gene that is in general expressed only in embryonic stages or in adult stem cells, may confirm the importance of an expressed telomerase in small intestine, where it is necessary to repair and maintain the turnover of the epithelium. Our results are also in accordance with another study showing that *TERT* Ala279Thr is normally expressed in esophageal cancers, contributing to chromosome instability (Zhang *et al*. 2014).

## Discussion

Using analysis of germline DNA from family members affected with SI-NETs and sporadic SI-NET patients, we identified a monoallelic mutation causing an amino acid substitution p.(Gly396Asp) in *MUTYH* that was significantly enriched among the patients affected with SI-NETs, compared to the controls. Thus, this mutation is a good candidate for a risk factor predisposing to the disease. Moreover, the *OGG1*-related results ought to be discussed here, because MUTYH and OGG1 proteins share functional properties. Although we have not reached statistical significance for enrichment of the mutation *OGG1* p.(Arg46Gln) in patients vs controls, this variant should also be considered in future studies. We performed an analysis of statistical power to assess whether a cohort of SI-NET patients being 10 times larger (i.e. equal number of patients and controls from EpiHealth cohort, and assuming the same frequency of *OGG1* p.(Arg46Gln) mutation in SI-NET patient cohort) would be sufficient. The odds ratios in this hypothetical situation would be about two and would have a strong statistical significance. This *OGG1*-related finding calls for an extended project delineating a role of both *MUTYH* and *OGG1* variants in the pathogenesis of the disease.

Interestingly, a recent report on a related disease, pancreatic neuroendocrine tumors (PAN-NETs), has described mutations in the *MUTYH* gene. Mutation p.(Gly396Asp) in *MUTYH*, along with other pathogenic variants in this gene, were reported. The alterations described in tumors were usually affecting both alleles, suggesting a complete functional inactivation of this gene (Scarpa *et al*. 2017). In our cohort, monoallelic *MUTYH* p.(Gly396Asp) mutation was detected in six patients. It was observed in the germline of two patients from distinct families and four apparently sporadic subjects (Table 3 and Supplementary Table 1). For two of these latter sporadic patients, the only studied tissue was blood. For the third sporadic patient (254), DNA extracted from blood and a PT was tested, and Sanger sequencing confirmed the presence of the heterozygous *MUTYH* p.(Gly396Asp) variant. The same monoallelic state was also detected in a metastasis from sporadic patient 105; the only tissue tested for this subject. Furthermore, previous analyses of gene copy number alterations in SI-NETs showed that chromosome 1p (where *MUTYH* is located) is rarely affected by tumor-specific deletions, which speaks against a frequent biallelic inactivation of this gene (Cunningham *et al*. 2011). These results might suggest that biallelic inactivation of *MUTYH* might not be the only mechanism driving the tumor development of SI-NETs, as opposed to what was observed in PAN-NETs (Scarpa *et al*. 2017). We therefore hypothesize that additional mutation(s) promoting the onset of the disease might occur in other genes related to excision-repair pathway and *OGG1* is a plausible candidate. This matter deserves follow-up studies for both SI-NETs and PAN-NETs.

*MUTYH* and *OGG1* are the orthologues of the bacterial *MutY* and *MutM* genes respectively, in *Escherichia coli* and are therefore highly evolutionarily conserved (Michaels & Miller 1992). They have a synergistic role in protecting DNA from oxidative damage. The protein product of *OGG1* is responsible for the excision of the mutagenic base 8-oxoguanine, while the enzyme encoded by *MUTYH* excises adenine bases at sites where they are mismatched with the wrong base. Both these mutational events occur normally in cells as a consequence of oxidative damage of DNA (Michaels & Miller 1992). A large number of various biallelic recessive mutations in *MUTYH* in the germline of patients with colorectal adenomatous polyposis have been described. Furthermore, these mutations have also been associated with extra-colonic tumors in subjects bearing two mutations in this gene. In rare cases, SI-NETs have been shown to be part of the clinical spectrum of patients with colorectal tumors. Notably, amino acid substitution p.(Gly396Asp) *MUTYH* is among the recessive germline mutations described so far in the *MUTYH* gene and likely causing the autosomal recessive form of adenomatous polyposis (Al-Tassan *et al*. 2002, Sampson *et al*. 2003, Sieber *et al*. 2003, Vogt *et al*. 2009). Moreover, the link between impaired function of *MUTYH* and *OGG1* and various tumors has been shown in mice bearing biallelic knockouts of these genes (Xie *et al*. 2004, Sakamoto *et al*. 2007). Interestingly, deficient excision of the mutagenic base 8-oxoguanine causes mutations in codon 12 of *k-ras* gene in mice (Xie *et al*. 2004) and codon 12 mutations in human *K-RAS* have also been described as a somatic tumor-specific change in SI-NETs (Banck *et al*. 2013).

Considering the above, we hypothesize that a monoallelic mutation causing amino acid substitution p.(Gly396Asp) in *MUTYH* is conferring a mild functional impairment, affecting the excision-repair system of 8-oxoguanine (without disturbing the level of MUTYH protein expression), eventually leading to the development of SI-NETs. Such a mutation might set up an environment that is less protected from reactive oxygen species. The presence of one copy of the wild-type allele in patients with *MUTYH* p.(Gly396Asp) might temper the effect of the altered protein, giving SI-NET patient a mild phenotype, late onset and slow progression of the disease. We speculate that in the presence of the biallelic *MUTYH* mutation (as is the case for autosomal recessive form of adenomatous polyposis (Al-Tassan *et al*. 2002, Sampson *et al*. 2003, Sieber *et al*. 2003, Vogt *et al*. 2009)), one might, for most of patients, never have time to observe the onset of SI-NETs, since other aggressive pathologies (such as colorectal cancer) might lead to the death of the patient. In this context, one could mention that heterozygous variant p.(Gly396Asp) in the MutY DNA glycosylase gene has been shown to be associated with an elevated risk of breast cancer also in a study on 930 Sephardi Jewish women of North African origin (Rennert *et al*. 2012). The p.(Gly396Asp) in *MUTYH* is, however, unlikely a single and sufficient event to cause the development of SI-NETs. Other mutations in additional members of the same excision-repair pathway might also play a role, as well as genes that are not related to this pathway.

The observed variants in *TERT* as well as in *SDHA*, *SDHB* and *SDHD* genes are actually the most common findings in our familial SI-NET patients, but these alleles are not enriched in frequency among all SI-NET patients, when compared to the control population. Furthermore, mutations in the *SDHx* genes have previously been implicated in cancer, and particularly, in forms related to SI-NETs, such as pheochromocytoma and paraganglioma (Cascon *et al*. 2002*b*, Perren *et al*. 2002). Somatic, tumor-specific *SDHD* mutations have also been shown in SI-NETs (Kytola *et al*. 2002). At the current stage of exploration of the significance of these genes in SI-NET patients, it is not possible to draw a firm conclusion. A larger set of familial and sporadic patients should be studied. We may only speculate that the variants we characterized could contribute to the development of tumors in some patients or in some families. They perhaps might represent phenotype modifying events, co-operating with mutations in other genes for the phenotype to appear, but not being by themselves rate-limiting predisposing mutations. It is noteworthy that the *TERT*, *SDHA*, *SDHB* and *SDHD* genes are all involved in pathways related to generation of and response to oxidative stress (Saretzki 2009).

Our finding of statistically significant difference in age at diagnosis for familial and sporadic SI-NET patients is consistent with the presence of an inherited component among the members of families affected with this disease. However, this presumed inherited component behind familial SI-NETs appears complex and unclear. The only previously characterized mutation (4-bp deletion in the *IPMK* gene) predisposing to familial SI-NETs is apparently a private mutation, specific for one large family (Sei *et al*. 2015). Mutations in *IMPK* could be found neither in the remaining set of 32 families studied by Sei and coworkers nor in the set of 15 families studied here. Furthermore, all but one so far reported families with patients affected by clinically diagnosed SI-NETs are unsuitable for linkage studies, in order to delineate a location of a gene predisposing for the disease. These families might rather be categorized as familial clusters and usually involve two affected subjects in one or two generations, that is kindreds without clear Mendelian inheritance. Moreover, in the large family reported by Sei and coworkers, only two individuals (out of 14 in the third generation with the *IPMK* gene mutation, Fig. 2 in ref.: (Sei *et al*. 2015)) were diagnosed with an SI-NET prior to advanced clinical screening. Another six individuals were diagnosed after screening procedures including PET examination with L-DOPA and/or capsule endoscopy and subsequent surgery; i.e. about 43% of clinically asymptomatic subjects (with the mutation) in this family had occult tumors, which is a very high number. Thus, it appears that expressivity of the *IPMK* mutation in causing SI-NETs is variable and there are likely additional mutation carriers without clinical symptoms of the disease. In this context, one should also discuss the important carcinoid (SI-NET) study from Southern Sweden based on autopsies. It clearly showed that the majority (90%) of subjects studied post mortem and having SI-NETs represented clinically silent tumors (Berge & Linell 1976). Consequently, various alleles that may predispose to the familial form of this disease might be relatively common in the general population. This suggests, therefore, that perhaps the biggest challenge in the field of genetics behind SI-NETs is to delineate how large fraction of SI-NET patients, which are assessed clinically as sporadic cases, might actually have a familial background.

In conclusion, SI-NETs can present as both a sporadic and a familial disease. We have identified monoallelic germline mutations in *MUTYH*, involved in DNA repair following oxidative stress, which may be a candidate gene for predisposition to SI-NET. However, further studies with a larger number of families and sporadic patients are needed in order to better understand the genetics behind the development of familial SI-NETs.

## Supplementary Material

Supporting Figure 1

Supporting Figure 2

Supporting Figure 3

Supporting Figure 4

Supporting Figure 5

Supporting Figure 6

Supporting Figure 7

Supporting Table 1

Supporting Table 2

## Declaration of interest

J P D and L A F are co-founders and shareholders in Cray Innovation AB.

## Funding

This study was supported by the grants from the Swedish Cancer Society, the Swedish Research Council, the Torsten Söderberg Foundation and Sci-Life-Lab-Uppsala to J P D and by the grants from the Swedish Cancer Society, Lion’s Cancer foundation at Uppsala University Hospital and the Selander’s foundation to E T J. Genotyping and next-generation sequencing were performed by the SNP&SEQ Technology Platform in Uppsala, Sweden, which is part of Science for Life Laboratory at Uppsala University and supported as a national infrastructure by the Swedish Research Council.

## Author contribution statement

Study design: J P D, C R, E T J. Interpretation of data: J D P, C R, H D, L A F, E T J. Laboratory work: C R, P B, H D, A S A, M G, J L C. Collection of patient and control samples and clinical information: S W, H S, H G, J L C, L L, E I, P S, P H, E T J. Writing the manuscript: J P D, C R, E T J. All co-authors were reading and commenting on the manuscript.
