# Supplementary figures and images for: A MUTYH germline mutation is associated with small intestinal neuroendocrine tumors
Source: Endocr Relat Cancer. 2017 Jun 20;24(8):427–43. doi: 10.1530/ERC-17-0196 (PMC5527373; doi:10.1530/ERC-17-0196)

Supplementary Figure 1.

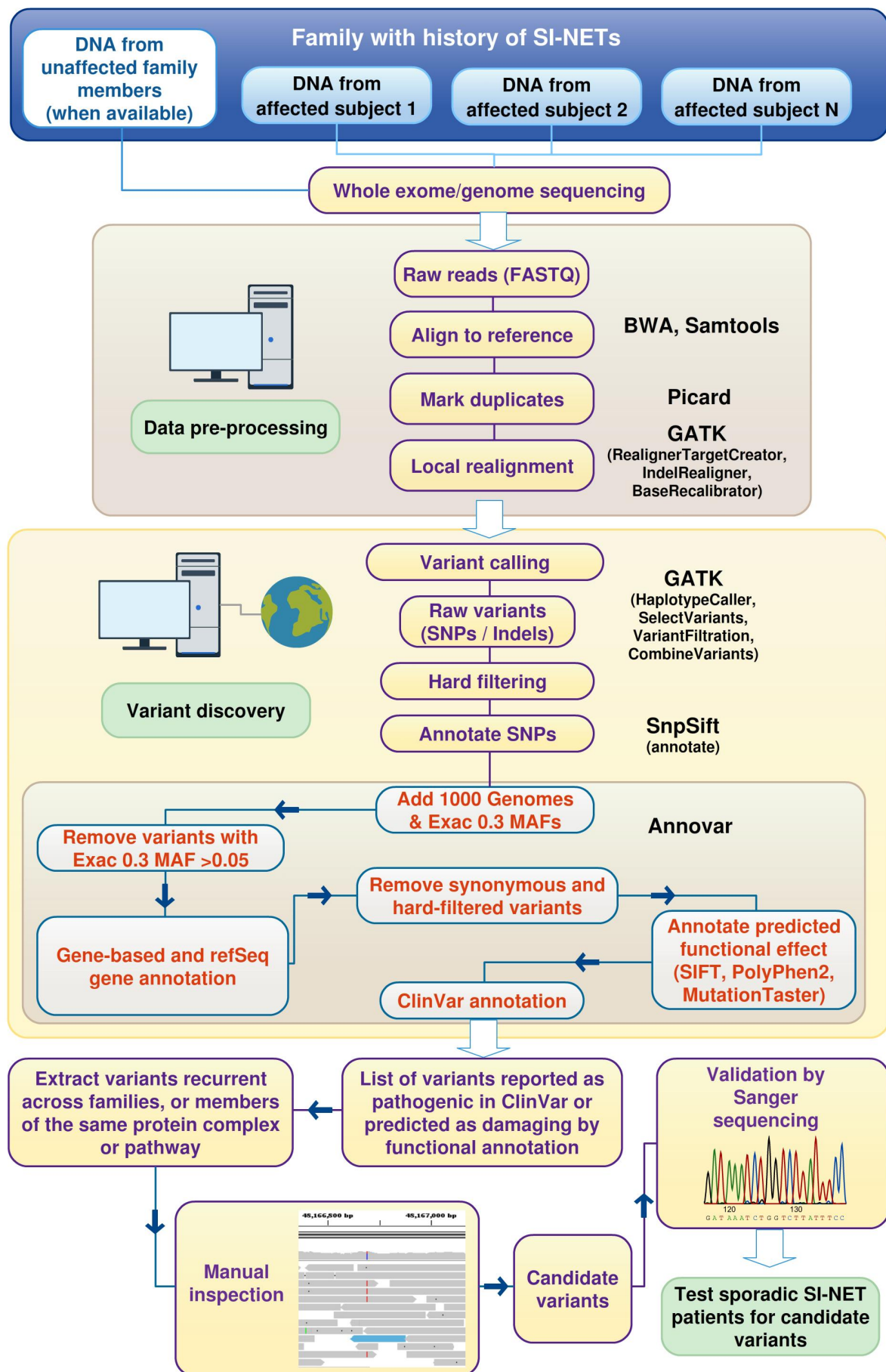

Supplement: Supporting Figure 1 [file erc-24-427-s001.pdf]

Supplementary Figure 2.

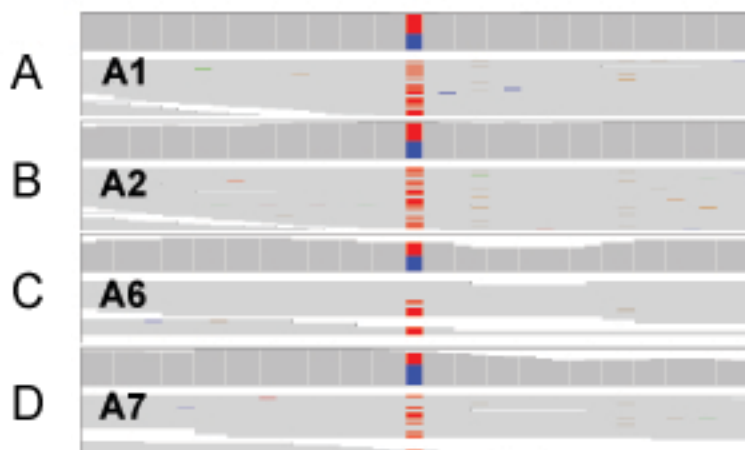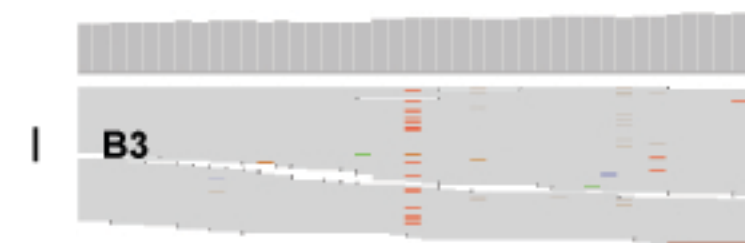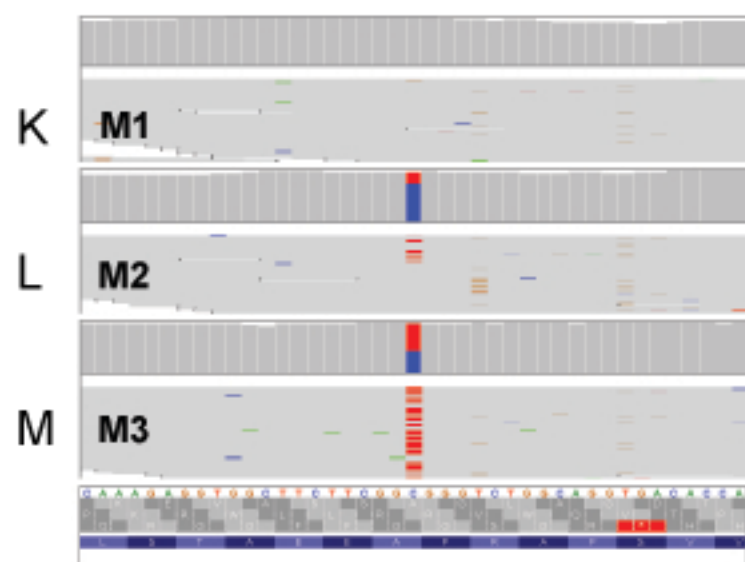

chr5:1294146-1294186

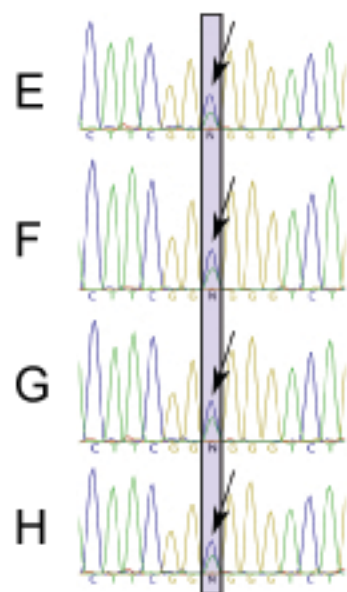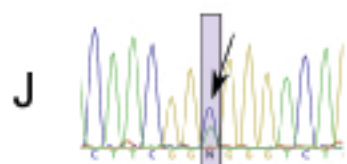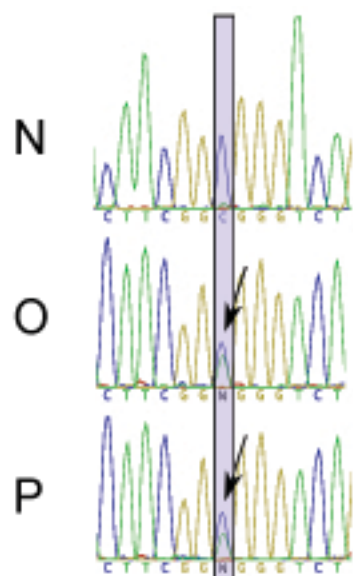

NM\_198253.2 (*TERT*):  
c.835G>A p.(Ala279Thr)

Supplement: Supporting Figure 2 [file erc-24-427-s002.pdf]

Supplementary Figure 3.

A

G1

B

G2

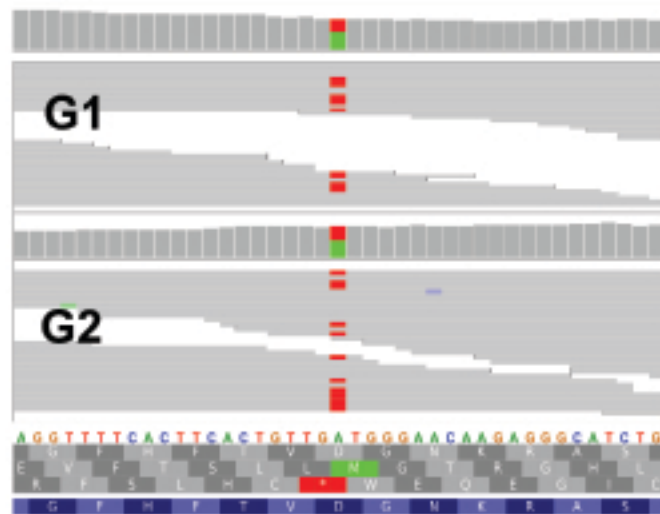

C

D

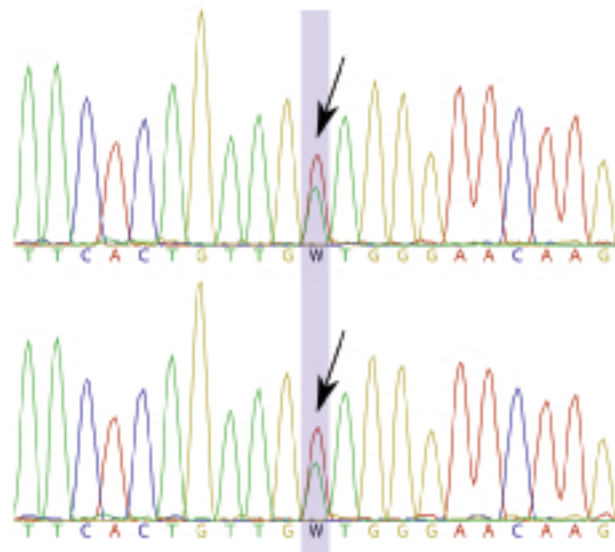

NM\_004168.3(*SDHA*):  
c.113A>T p.(Asp38Val)

Supplement: Supporting Figure 3 [file erc-24-427-s003.pdf]

Supplementary Figure 4.

A

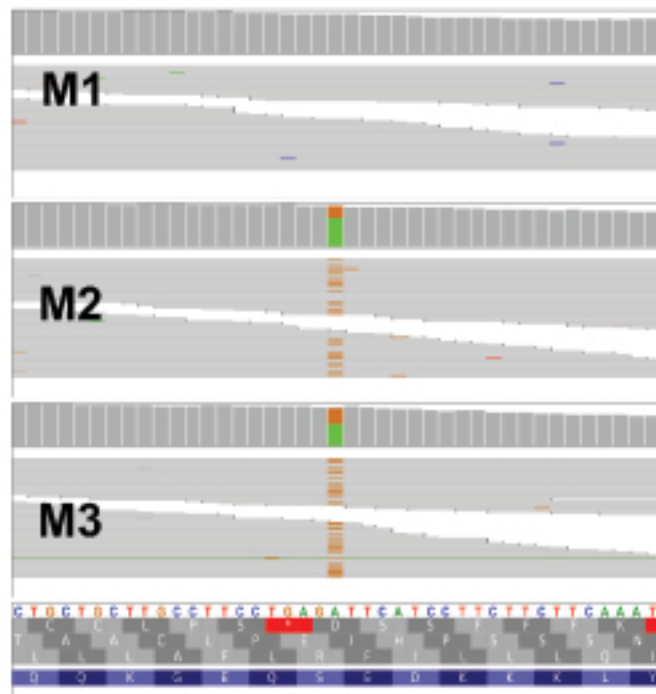

B

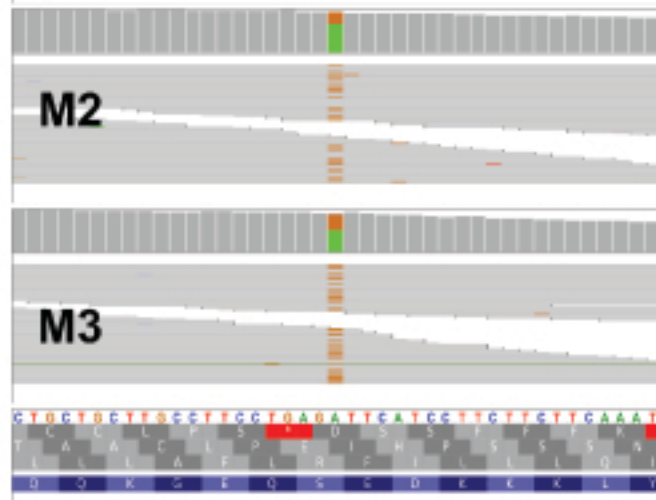

C

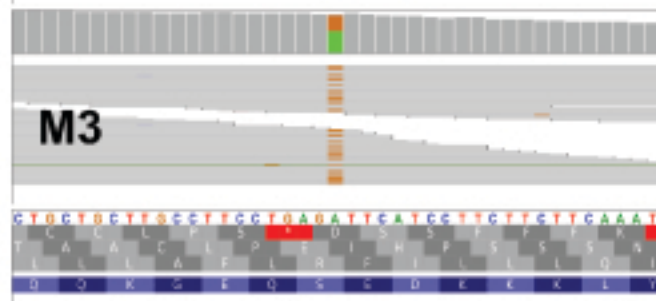

chr1:17354277-17354317

D

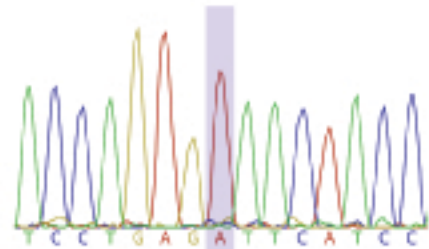

E

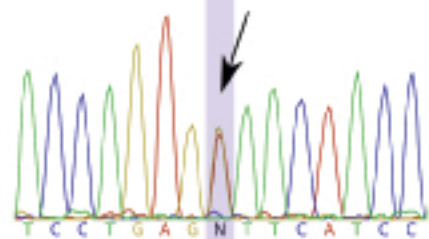

F

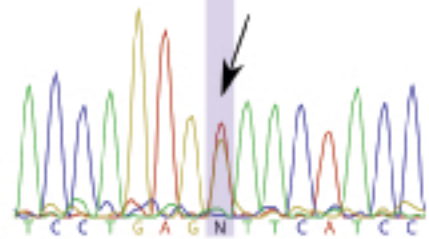

NM\_003000.2(*SDHB*):  
c.487T>C p.(Ser163Pro)

Supplement: Supporting Figure 4 [file erc-24-427-s004.pdf]

Supplementary Figure 5.

A

D8

B

D9

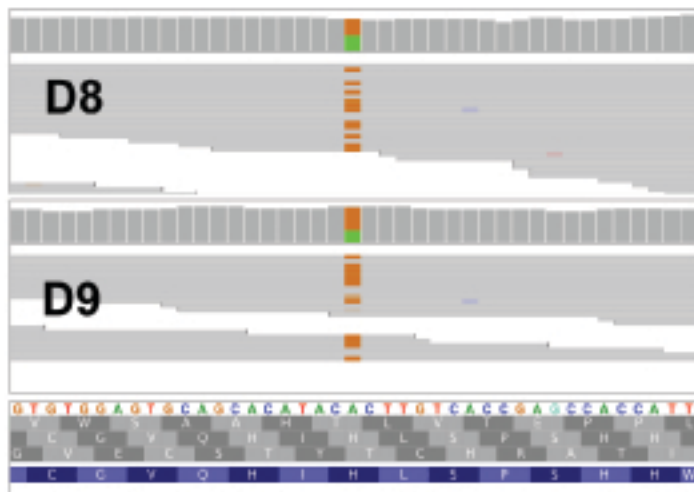

chr11:111958657-111958697

C

D

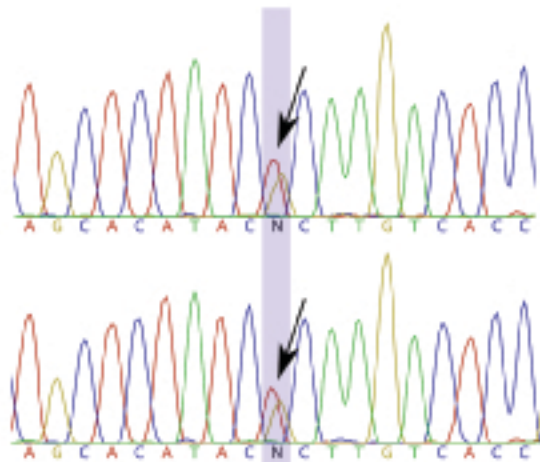

NM\_003002.3(*SDHD*):  
c.149A>G p.(His50Arg)

Supplement: Supporting Figure 5 [file erc-24-427-s005.pdf]

Supplementary Figure 6.

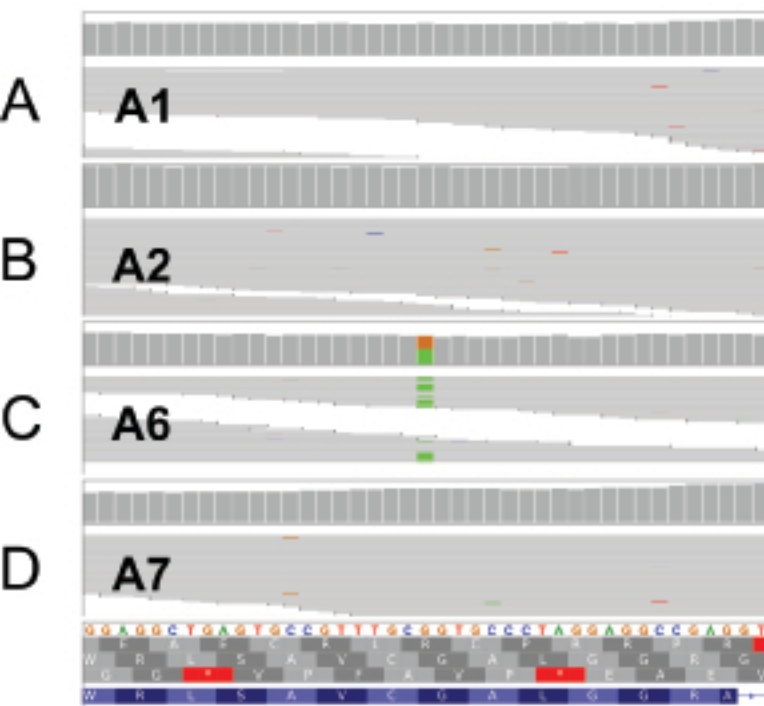

chr11:111957645-111957685

**E**

**F**

**G**

**H**

NM\_003002.3(*SDHD*):  
c.34G>A p.(Gly12Ser)

Supplement: Supporting Figure 6 [file erc-24-427-s006.pdf]

Supplementary Figure 7

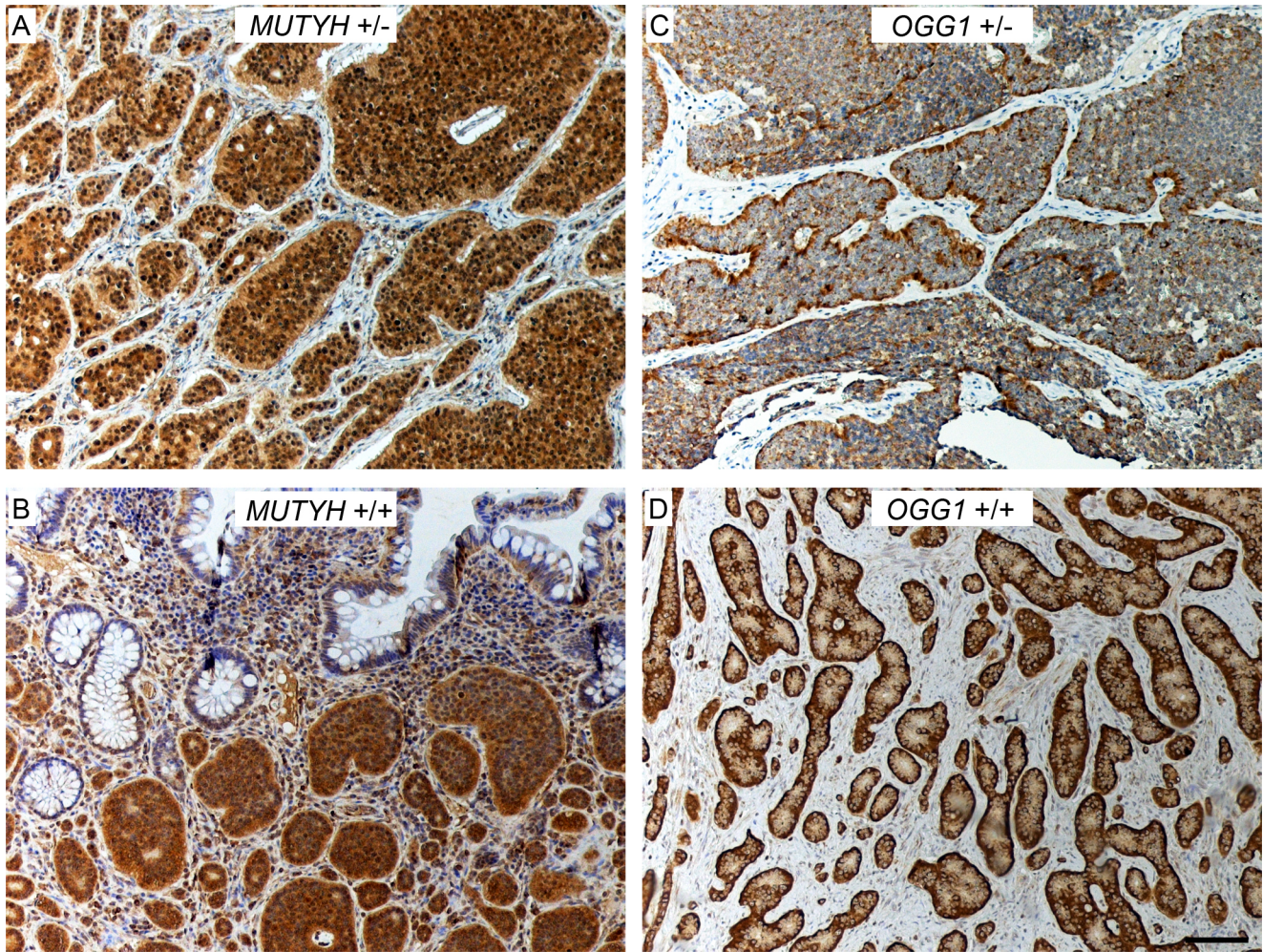

Supplement: Supporting Figure 7 [file erc-24-427-s007.pdf]
