# Supplementary material for: A MUTYH germline mutation is associated with small intestinal neuroendocrine tumors
Source: Endocr Relat Cancer. 2017 Jun 20;24(8):427–43. doi: 10.1530/ERC-17-0196 (PMC5527373; doi:10.1530/ERC-17-0196)
Supplement: Supporting Table 1 [file erc-24-427-t001.pdf]

Supplementary Table 1. Summary of clinical data and Sanger sequencing detection of seven candidate variants in DNA samples from sporadic SI-NET patients.

									Candidate variants tested by Sanger sequencing[g]						
ID[a]	Sex	Tissue[b]	Age at diagn.	Ki-67[c]	WHO[d]	Tumor Stage[e]	Age at Death[f]	Survival (months)	*MUTYH* p. (Gly396Asp)	*OGG1* p. (Arg46Gln)	*SDHA* p. (Asp38Val)	*SDHB* p. (Ser163Pro)	*SDHD* p. (Gly12Ser)	*SDHD* p. (His50Arg)	*TERT* p. (Ala279Thr)
1	M	B	n.a.	1%	1	TxN1M1	63	n.a.					+/-		
1	M	M (liver)	n.a.	1%	1	TxN1M1	63	n.a.					+/-		
2	M	B	n.a.	1%	1	TxN1M1	AWD	n.a.							
3	M	B	n.a.	9%	2	TxN1M1	74	n.a.							
3	M	PT	n.a.	9%	2	TxN1M1	74	n.a.							
4	F	B	n.a.	0.1%	1	TxN1M0	AWD	n.a.							
4	F	PT	n.a.	0.1%	1	TxN1M0	AWD	n.a.							
5	F	B	n.a.	8%	2	TxN1M0	AWD	n.a.							
5	F	PT	n.a.	8%	2	TxN1M0	AWD	n.a.		n.a.					
6	F	B	61	n.a.	n.a.	TxN1M1	73	139			+/-				
7	F	B	62	10%	2	TxN1M1	70	92							
8	F	B	52	n.a.	n.a.	TxN1M1	64	146							
9	F	B	61	<1%	1	TxN1M0	AWD	168							
10	F	B	55	n.a.	n.a.	TxN1M1	66	126							
11	M	B	69	n.a.	n.a.	TxN1M0	89	238							+/-
12	F	B	59	1-2%	1	TxN1M0	70	126						+/-	
13	F	B	56	<1%	1	TxN1M1	AWD	249							
14	M	B	53	<1%	1	TxN1M1	AWD	160							
15	M	B	63	n.a.	n.a.	TxN1M0	81	216							
16	F	B	64	2%	1	TxN1M1	72	90							
17	F	B	62	1%	1	TxN1M1	AWD	145							
18	F	B	73	1%	1	TxN1M1	78	60							
19	M	B	72	10%	2	TxN1M1	77	55		+/-		+/-			
20	F	B	54	<1%	1	TxN1M1	AWD	224							+/-
20	F	M (colon)	54	<1%	1	TxN1M1	AWD	224							+/-
21	M	B	62	1%	1	TxN1M0	74	143							
22	F	B	55	<1%	1	TxN1M1	67	151							
23	F	B	69	<1%	1	TxN1M0	AWD	174							
24	F	B	45	1%	1	TxN1M1	62	207							
25	M	B	55	0.50%	1	TxN1M1	AWD	215							
26	F	B	69	1%	1	TxN1M0	77	89							
27	M	B	73	<1%	1	TxN1M0	AWD	169							
28	M	B	84	<1%	1	TxN1M1	86	25							
29	M	B	64	1-2%	1	TxN1M0	AWD	147							
29	M	M (meso)	64	1-2%	1	TxN1M0	AWD	147							
30	M	B	60	<1%	1	TxN1M1	73	164							+/-
31	M	B	63	n.a.	n.a.	TxN1M1	81	211							
32	F	B	69	n.a.	n.a.	TxN1M0	AWD	195							
33	M	B	40	n.a.	n.a.	TxN1M1	AWD	348							
34	M	B	62	<1%	1	TxN1M1	AWD	141							
35	F	B	54	2%	1	TxN1M1	90	426							
36	M	B	69	<1%	1	TxN1M1	77	97							+/-

37	F	B	50	<1%	1	TxN1M1	59	104							
37	F	M (liver)	50	<1%	1	TxN1M1	59	104							
37	F	NT (liver)	50	<1%	1	TxN1M1	59	104							
38	F	B	52	<1%	1	TxN1M1	AWD	214			+/-				
38	F	PT	52	<1%	1	TxN1M1	AWD	214			+/-				
39	F	B	57	1%	1	TxN1M1	66	103							
40	F	B	49	1-2%	1	TxN1M0	AWD	181							
40	F	M	49	1-2%	1	TxN1M0	AWD	181							
41	M	B	70	n.a.	n.a.	TxN1M1	AWD	243					+/-		
42	M	B	38	n.a.	n.a.	TxN1M1	58	244							
43	M	B	40	4%	2	TxN1M1	54	171							
44	M	B	56	1-2%	1	TxN1M1	AWD	138							
45	M	B	71	5-10%	2	TxN1M1	72	18							
46	M	B	49	<1%	1	TxN1M1	72	277							
47	M	B	62	<1%	1	TxN1M1	AWD	154							
48	F	B	52	10%	2	TxN1M1	52	8							
49	M	B	52	n.a.	n.a.	TxN1M1	58	82			+/-				
50	M	B	59	n.a.	n.a.	TxN1M1	70	126							
51	M	B	66	n.a.	n.a.	TxN1M0	AWD	156	+/-						
52	M	B	68	1%	1	TxN1M1	AWD	141							
53	M	B	61	2%	1	TxN1M0	AWD	140							
54	M	B	66	n.a.	n.a.	TXn1m0	74	101							
55	F	B	67	1%	1	TxN1M1	AWD	178							
55	F	NT	67	1%	1	TxN1M1	AWD	178							
55	F	PT	67	1%	1	TxN1M1	AWD	178							
56	M	B	25	n.a.	n.a.	TxN1M1	AWD	236						+/-	
56	M	PT	25	n.a.	n.a.	TxN1M1	AWD	236						+/-	
57	M	B	52	1%	1	TxN1M1	AWD	176							
58	F	B	40	1%	1	TxN1M1	AWD	183							
59	F	B	84	1%	1	TxN1M0	AWD	150							
60	F	B	77	3%	2	TxN1M1	AWD	123	+/-						
61	F	B	76	2%	1	TxN1M0	84	99							
62	F	B	59	<1%	1	TxN1M1	72	152							
62	F	M (meso)	59	<1%	1	TxN1M1	72	152							
64	F	B	52	1%	1	TxN1M1	AWD	141							
64	F	M (liver)	52	1%	1	TxN1M1	AWD	141							
65	M	B	49	1%	1	TxN1M1	AWD	161							
66	F	B	59	5%	2	TxN1M1	69	120							
67	F	B	42	2%	1	TxN1M1	58	193							
68	M	B	62	5%	2	TxN1M1	81	235			+/-				
68	M	M (liver)	62	5%	2	TxN1M1	81	235			+/-				
69	F	B	49	2%	1	TxN1M0	AWD	137							+/-
70	M	B	59	<1%	1	TxN1M1	68	111							
72	F	B	66	n.a.	n.a.	TxN1M0	82	196							+/-
73	F	B	38	n.a.	n.a.	TxN1M0	58	232							
74	M	B	56	n.a.	n.a.	TxN1M0	AWD	220				+/-			
75	M	B	54	1-2%	1	TxN1M1	AWD	182					+/-		
75	M	M	54	1-2%	1	TxN1M1	AWD	182					+/-		
76	M	B	46	1%	1	TxN1M0	AWD	160				+/-			

77	M	B	78	5%	2	TxN1M0	85	82							
78	M	B	57	1%	1	TxN1M1	73	194							
79	F	B	65	1%	1	TxN1M0	68	35							
80	M	B	46	1-2%	1	TxN1M1	54	102							
81	F	B	59	0.5%	1	TxN1M0	AWD	141							
82	F	B	57	1%	1	TxN1M0	AWD	123							
83	M	B	56	<3%	2	TxN1M1	64	93							
84	F	B	78	6%	2	TxN1M0	80	25							
85	M	B	54	6-9%	2	TxN1M1	61	90							
86	F	B	46	1%	1	TxN1M1	66	235							
87	M	B	59	2-3%	1	TxN1M0	AWD	177							
88	M	B	47	1%	1	TxN0M0	51	51							
89	M	B	62	1%	1	TxN1M1	74	141							
90	F	B	72	<1%	1	TxN1M0	83	135							+/-
91	F	B	72	1%	1	TxN1M0	84	142							
92	F	B	68	n.a.	n.a.	TxN1M0	AWD	231							
93	M	B	64	0.1%	1	TxN0M1	AWD	135			+/-				
94	F	B	51	2-3%	2	TxN1M0	AWD	133							
95	M	B	42	1-2%	1	TxN1M1	AWD	139							
96	F	B	45	1%	1	TxN1M1	AWD	172							
97	F	B	67	1%	1	TxN1M1	80	155							
97	F	M (liver)	67	1%	1	TxN1M1	80	155							
97	F	NT	67	1%	1	TxN1M1	80	155							
97	F	PT	67	1%	1	TxN1M1	80	155							
98	F	B	72	1%	1	TxN1M1	AWD	132			+/-				
99	M	B	53	1%	1	TxN1M1	AWD	204							
100	M	B	67	1%	1	TxN1M1	AWD	127				+/-			
101	F	B	72	n.a.	n.a.	TxN1M1	86	168							
102	F	B	60	2%	1	TxN1M1	68	101							
104	F	M	44	n.a.	n.a.	TxN1M1	60	197							
105	M	M (liver)	n.a.	n.a.	n.a.	n.a.	n.a.	n.a.	+/-		+/-				
106	F	M (liver)	48	n.a.	n.a.	TxN1M0	60	143							
107	F	PT	62	n.a.	n.a.	TxN1M1	73	130							
108	M	PT	78	n.a.	n.a.	TxN1M1	81	45							
109	M	M (liver)	57	n.a.	n.a.	TxN1M1	60	45							
110	F	PT	63	n.a.	n.a.	TxN1M1	66	36							
111	M	PT	90	n.a.	n.a.	TxN1M0	104	167							+/-
112	M	NT	58	n.a.	n.a.	TxN1M1	AWD	374							
112	M	PT	58	n.a.	n.a.	TxN1M1	AWD	374	n.a.						n.a.
113	F	PT	61	n.a.	n.a.	TxN1M1	67	78			+/-				
115	F	PT	69	n.a.	n.a.	TxN1M0	72	34							
116	M	NT	56	6%	2	TxN1M1	67	134							
116	M	PT	56	6%	2	TxN1M1	67	134							
117	M	NT (liver)	70	n.a.	n.a.	TxN1M1	84	159							
117	M	PT	70	n.a.	n.a.	TxN1M1	84	159							
118	F	PT	61	n.a.	n.a.	TxN1M1	76	177							
121	M	M	n.a.	n.a.	n.a.	n.a.	n.a.	n.a.							
122	M	PT	75	n.a.	n.a.	TxN1M0	87	143							
123	F	M (liver)	67	n.a.	n.a.	TxN1M1	72	63							

124	F	M	59	n.a.	n.a.	TxN1M1	58	1		+/-		
125	F	NT (liver)	46	1%	1	TxN1M1	AWD	264				
125	F	PT	46	1%	1	TxN1M1	AWD	264				
126	M	M (liver)	72	n.a.	n.a.	TxN1M1	80	98				
126	M	PT	n.a.	n.a.	n.a.	NULL	n.a.	n.a.				
127	M	PT	n.a.	n.a.	n.a.	NULL	n.a.	n.a.				
129	F	PT	65	n.a.	n.a.	TxN1M1	68	39				
130	F	PT	74	n.a.	n.a.	TxN1M1	75	12				n.a.
131	F	PT	49	n.a.	n.a.	TxN1M1	52	38				
132	M	M (meso)	76	n.a.	n.a.	TxN1M1	80	54				
134	M	PT	46	2%	1	TxN1M1	52	69				
135	M	PT	66	n.a.	n.a.	TxN1M1	67	13		+/-		n.a.
137	F	NT	63	n.a.	n.a.	TxN1M0	66	33			n.a.	n.a.
137	F	M (omentum)	63	n.a.	n.a.	TxN1M0	66	33				
138	M	M (liver)	41	n.a.	n.a.	TxN1M1	46	60				
138	M	PT	41	n.a.	n.a.	TxN1M1	46	60				
139	M	NT	69	n.a.	n.a.	TxN0M0	76	86		+/-		
139	M	PT	69	n.a.	n.a.	TxN0M0	76	86		+/-		
141	M	M (meso)	39	1%	1	TxN1M1	52	155				
143	M	PT	59	n.a.	n.a.	TxN1M1	62	39				
146	F	M (meso)	55	n.a.	n.a.	TxN1M0	AWD	216				
147	F	M	40	1%	1	TxN1M1	57	200				n.a.
147	F	NT	40	1%	1	TxN1M1	57	200				
148	M	M (meso)	48	1%	1	TxN1M1	50	29				n.a.
149	F	ANT	73	n.a.	n.a.	TxN1M1	87	167				
149	F	PT	73	n.a.	n.a.	TxN1M1	87	167				
151	M	PT	61	n.a.	n.a.	TxN1M1	70	102				
152	M	PT	70	1%	1	TxN1M0	82	146				
154	M	M (liver)	64	n.a.	n.a.	TxN1M0	81	210				
154	M	NT (liver)	64	n.a.	n.a.	TxN1M0	81	210				
155	M	M (meso)	69	1%	1	TxN1M1	71	28				+/-
156	F	PT	60	n.a.	n.a.	TxN1M1	63	32				
161	F	PT	70	n.a.	n.a.	TxN1M1	76	66				
165	M	PT	71	<1%	1	TxN1M0	74	115				
166	F	PT	44	1%	1	TxN1M1	AWD	224				
168	F	PT	68	20%	2	TxN1M1	70	34				
169	M	PT	70	<1%	1	TxN1M1	74	51				
170	F	PT	74	1%	1	TxN1M1	88	163				
171	M	PT	51	3%	2	TxN1M1	64	148				
173	M	M (liver)	52	n.a.	n.a.	TxN1M1	56	51				
173	M	NT	52	n.a.	n.a.	TxN1M1	56	51				
175	F	M (ovaries)	78	1%	1	TxN1M1	78	2				
176	M	M (meso)	67	<1%	1	TxN1M1	AWD	161				
176	M	NT (liver)	67	<1%	1	TxN1M1	AWD	161				n.a.
179	M	NT	57	<1%	1	TxN1M1	AWD	144	n.a.			
179	M	PT	57	<1%	1	TxN1M1	AWD	144				n.a.
180	F	M (ovaries)	46	<1%	1	n.a.	70	282				
183	F	M (meso)	66	1%	1	TxN1M1	AWD	136				
184	M	PT	70	1%	1	TxN1M1	78	94				

185	F	M (meso)	57	1%	1	TxN1M0	77	238							
187	F	PT	76	1%	1	TxN1M1	85	98							n.a.
188	F	M (peritoneum)	42	1%	1	TxN1M0	AWD	252							n.a.
191	F	PT	66	3%	1	TxN1M1	69	34							n.a.
192	M	M (meso)	70	<1%	1	TxN1M0	79	111							
193	M	M (meso)	46	1%	1	TxN1M0	AWD	213							
194	F	NT (liver)	51	2-3%	2	TxN1M1	AWD	116							
194	F	PT	51	2-3%	2	TxN1M1	AWD	116							n.a.
195	M	M (meso)	65	1%	1	TxN1M1	AWD	114							
197	M	M	40	1%	1	TxN1M0	AWD	154		+/-					n.a.
198	M	M (meso)	67	1%	1	TxN1M1	AWD	110							
199	F	PT	70	1%	1	TxN1M0	77	77							
200	M	M (meso)	71	1%	1	TxN1M0	AWD	104							
202	M	NT (liver)	60	1-2%	1	TxN1M1	AWD	108		+/-					
202	M	PT	60	1-2%	1	TxN1M1	AWD	108	n.a.	+/-					
203	F	M	72	2-3%	2	TxN1M0	76	55							
203	F	NT	72	2-3%	2	TxN1M0	76	55							
205	F	NT	43	5%	2	TxN1M1	AWD	100							
205	F	PT	43	5%	2	TxN1M1	AWD	100							
207	F	NT	57	10%	2	TxN1M1	AWD	94							
207	F	PT	57	10%	2	TxN1M1	AWD	94							
208	M	M (meso)	55	2.5%	1	TxN1M0	AWD	89			+/-				
208	M	NT	55	2.5%	1	TxN1M0	AWD	89			+/-				
209	F	PT	72	<1%	1	TxN1M1	AWD	82							
210	F	M (ovaries)	62	3%	2	TxN1M1	66	48							
210	F	NT	62	3%	2	TxN1M1	66	48							
211	M	PT	72	1%	1	TxN1M0	AWD	83			+/-				
212	F	M (liver)	41	1%	1	TxN1M1	AWD	84							
213	F	M (ovaries)	40	2-3%	1	TxN1M1	AWD	86			+/-				n.a.
214	M	ANT	55	<1%	1	TxN1M1	AWD	81		n.a.					
214	M	M	55	<1%	1	TxN1M1	AWD	81							
215	F	NT (pancreas)	72	2-3%	2	TxN1M0	78	71			+/-				
215	F	PT	72	2-3%	2	TxN1M0	78	71			+/-				
217	M	NT (liver)	66	1-3%	2	TxN1M0	AWD	77							
217	M	PT	66	1-3%	2	TxN1M0	AWD	77							
218	M	M (peritoneum)	69	<1%	1	TxN1M1	77	89							+/-
219	M	NT (liver)	66	1%	1	TxN1M1	AWD	74							
219	M	PT	66	1%	1	TxN1M1	AWD	74							
221	F	NT	58	1%	1	TxN1M1	AWD	78							
221	F	PT	58	1%	1	TxN1M1	AWD	78							
222	M	PT	43	2%	1	TxN1M1	AWD	74							
223	F	M (meso)	64	4%	2	TxN1M1	AWD	94							
224	M	M (meso)	68	3%	2	TxN1M0	AWD	66							
225	M	M	64	15%	2	TxN1M1	AWD	73							
227	M	NT	68	2-3%	2	TxN1M1	70	19							
227	M	PT	68	2-3%	2	TxN1M1	70	19							
229	F	M (ovaries)	66	7%	2	TxN1M1	69	39							
230	M	M (meso)	44	10%	2	TxN1M1	AWD	51							
232	F	B	67	1%	1	TxN1M1	AWD	32							

232	F	NT	67	1%	1	TxN1M1	AWD	32							
233	M	B	67	2%	1	TxN1M1	AWD	34							
233	M	M (meso)	67	2%	1	TxN1M1	AWD	34							
233	M	NT (liver)	67	2%	1	TxN1M1	AWD	34							
233	M	PT	67	2%	1	TxN1M1	AWD	34							
235	M	B	59	2.7%	2	TxN1M1	AWD	33							
235	M	PT	59	2.7%	2	TxN1M1	AWD	33	n.a.						n.a.
236	M	B	59	1%	1	TxN1M1	AWD	32							
236	M	M (meso)	59	1%	1	TxN1M1	AWD	32							
236	M	NT (lever)	59	1%	1	TxN1M1	AWD	32							
236	M	PT	59	1%	1	TxN1M1	AWD	32							
238	M	B	68	17%	2	TxN1M0	AWD	29							
238	M	M (meso)	68	17%	2	TxN1M0	AWD	29							
238	M	PT	68	17%	2	TxN1M0	AWD	29							
239	F	B	53	15%	2	TxN1M1	AWD	28							
239	F	M (omentum)	53	15%	2	TxN1M1	AWD	28							
239	F	PT	53	15%	2	TxN1M1	AWD	28							
240	M	B	71	2-3%	2	TxN1M0	AWD	29							
240	M	M (meso)	71	2-3%	2	TxN1M0	AWD	29							
240	M	PT	71	2-3%	2	TxN1M0	AWD	29							
241	M	B	71	6%	2	TxN1M1	AWD	28							
241	M	PT	71	6%	2	TxN1M1	AWD	28				+/-		+/-	
242	F	B	23	1%	1	TxN1M1	AWD	28							
242	F	PT	23	1%	1	TxN1M1	AWD	28							n.a.
243	F	B	66	<1%	1	TxN1M1	AWD	176							
243	F	M (ovaries)	66	<1%	1	TxN1M1	AWD	176							
244	M	B	60	4%	2	TxN1M1	AWD	30							
244	M	M (liver)	60	4%	2	TxN1M1	AWD	30							
244	M	PT	60	4%	2	TxN1M1	AWD	30							
245	M	B	61	<1%	1	TxN1M1	AWD	26							
245	M	M (appendix)	61	<1%	1	TxN1M1	AWD	26	n.a.						
245	M	PT	61	<1%	1	TxN1M1	AWD	26							
246	F	B	63	<1%	1	TxN1M1	65	30							
246	F	PT	63	<1%	1	TxN1M1	65	30		n.a.					
247	M	B	78	3%	2	TxN1M1	AWD	49							
247	M	PT	78	3%	2	TxN1M1	AWD	49		n.a.					
249	M	B	61	5%	2	TxN1M1	AWD	43							
249	M	PT	61	5%	2	TxN1M1	AWD	43		n.a.					
250	M	B	75	3%	2	TxN1M0	AWD	36							
250	M	PT	75	3%	2	TxN1M0	AWD	36		n.a.					
251	M	B	68	<1%	1	TxN1M0	AWD	34							
251	M	PT	68	<1%	1	TxN1M0	AWD	34		n.a.					
252	M	B	73	<1%	1	TxN1M0	74	9			n.a.				
252	M	PT	73	<1%	1	TxN1M0	74	9		n.a.	n.a.				
253	M	B	58	3%	2	TxN1M1	AWD	30							
253	M	PT	58	3%	2	TxN1M1	AWD	30		n.a.					
254	M	B	64	2.4%	1	TxN1M0	AWD	28	+/-						
254	M	PT	64	2.4%	1	TxN1M0	AWD	28	+/-	n.a.					
255	F	B	68	3.2%	2	TxN1M1	AWD	26							

255	F	PT	68	3.2%	2	TxN1M1	AWD	26	n.a.	
256	M	B	58	5%	2	TxN1M1	AWD	22		
256	M	PT	58	5%	2	TxN1M1	AWD	22	n.a.	
257	F	B	75	<1%	1	TxN1M1	AWD	26		+/-
257	F	PT	75	<1%	1	TxN1M1	AWD	26	n.a.	
258	F	B	59	5%	2	TxN1M1	AWD	24		
259	F	PT	60	9%	2	TxN1M1	62	20		

n.a. - data not available

[a] Same IDs denotes different samples taken from the same patient.

[b] Abbreviations: B, blood; NT, normal tissue; PT, primary tumor; M, metastasis. The specific tissue used for extraction of DNA for normal tissue and metastases, whenever available, is specified in parentheses.

[c] Antigen Ki-67 is an important marker for cellular proliferation and mitotic activity, predictor of SI-NET behavior and patient survival (Sokmensuer C, Gedikoglu G, Uzunalimoglu B. Importance of proliferation markers in gastrointestinal carcinoid tumors: a clinicopathologic study. Hepato-gastroenterol. 2001;48:720–723).

[d] Grade of tumor according to the World Health Organization (WHO) classification (WHO Classification of Tumours of the Digestive System, WHO/IARC Classification of Tumours, 4th Edition, Volume 3).

[e] Tumors classified according to the AJCC Cancer Staging Manual (7th ed.), 2009.

[f] AWD - Alive with disease

[g] +/- denotes the presence of the variant in only one of the two alleles. All candidate SNPs tested in the sporadic Si-NETs cohort were in fact heterozygous.
