# Supplementary material for: A MUTYH germline mutation is associated with small intestinal neuroendocrine tumors
Source: Endocr Relat Cancer. 2017 Jun 20;24(8):427–43. doi: 10.1530/ERC-17-0196 (PMC5527373; doi:10.1530/ERC-17-0196)
Supplement: Supporting Table 2 [file erc-24-427-t002.pdf]

Supplementary Table 2. PCR primers for amplifying genomic regions containing the seven candidate variants identified in familial SI-NETs.

Gene	rsID	cDNA	Amino acid residue change	Forward primer sequence	Reverse primer sequence	Annealing temperature	PCR fragment length (GRCh37 genome assembly)
TERT	rs61748181	C/T	p.(Ala279Thr)	5′-GTACACCGGGGGACAAGG-3′	5′-AGTGCCAGCCGAAGTCTG-3′	67°C	297 bp
SDHA	rs34635677	A/T	p.(Asp38Val)	5′-GAACAGTTTGCAAGGGGAAA-3′	5′-CCTTACCCCCTAAGCCAAAA-3′	60°C	233 bp
SDHB	rs33927012	T/C	p.(Ser163Pro)	5′-TGCCAGTTCCTCTCCAGAAT-3′	5′-CCTGGCATAGAGTGGACGAG-3′	60°C	359 bp
SDHD	rs11214077	A/G	p.(His50Arg)	5′-TGTTGCTTCGAACTCCAGTG-3′	5′-TGTCTGCCCAAAGGTGTAAA-3′	60°C	265 bp
SDHD	rs34677591	G/A	p.(Gly12Ser)	5′-TCACCCAGCATTTCCTCTTC-3′	5′-AACTCGTCAGCTCACAGCAA-3′	60°C	350 bp
MUTYH	rs36053993	G/A	p.(Gly396Asp)	5′-TCACTTACCTCCCCAAGGTG-3′	5′-AGCTCTGCCACCTGTGTTCT-3′	60°C	325 bp
OGG1	rs104893751	G/A	p.(Arg46Gln)	5′-TAAGGGTCGTGGTCCTTGTC-3′	5′-TACCCGTGCTTGTTTCCTCT-3′	67°C	370 bp
